# Effect of Canthaxanthin on Egg Yolk Quality of Huaixiang Laying Hens at Normal and High Temperature

**DOI:** 10.3390/foods14060950

**Published:** 2025-03-11

**Authors:** Yiping Song, Sumeng Yu, Xiaofeng Zhang, Weixin Huang, Suiyang Tao, Jie Chen, Xiaoyun Zhou, Mei Xiao, Lilong An

**Affiliations:** Department of Animal Science, College of Coastal Agricultural Sciences, Guangdong Ocean University, Zhanjiang 524088, China; songyiping21@163.com (Y.S.); ysmlx123@163.com (S.Y.); m19570034570@163.com (X.Z.); kjnrpd@163.com (W.H.); tsygd2022@163.com (S.T.); jiechen222@163.com (J.C.); m3168868712@163.com (X.Z.); xiao0812@126.com (M.X.)

**Keywords:** canthaxanthin, egg yolk quality, high temperature, poultry, laying hens

## Abstract

(1) Background: Thermal stress in Guangdong’s tropical/subtropical regions significantly compromises yolk quality in Huaixiang chickens. Canthaxanthin (CX), an effective feed additive, has been demonstrated to not only enhance the nutritional composition of egg yolks but also mitigate heat stress. This study systematically evaluates the effects of dietary CX supplementation on egg production rate and yolk nutritional components (e.g., amino acids, lipids, vitamin B2) in Huaixiang hens under both normal and high-temperature environments. (2) Methods: A factorial design was implemented, exposing hens to either thermoneutral (25 ± 2 °C, 65–75% RH) or high-temperature (32 ± 2 °C, 65–75% RH) conditions. Diets were supplemented with graded CX levels (0, 4, 6, 8, or 10 mg/kg) for 9 weeks. Laying performance and yolk nutritional profiles (amino acids, lipids, minerals, vitamin B_2_) were evaluated at 3-week intervals (3rd, 6th, and 9th weeks. (3) Results: Under normal temperature conditions, the addition of CX significantly enhanced the laying rate, relative yolk weight, yolk color score, lecithin (LEC) content, and it optimized the amino acid profile of the yolk. Under high-temperature conditions, the laying rate, yolk weight, yolk weight ratio, total amino acid content, yolk triglyceride (TG), LEC, and vitamin B_2_ (VB_2_) levels in Huaixiang chickens all decreased. However, supplementation with CX under high-temperature conditions effectively mitigated these adverse effects: the laying rate was restored to levels observed under normal temperature conditions, while the relative yolk weight, color score, TG, total cholesterol (TC), LEC, high-density lipoprotein cholesterol (HDL-C), low-density lipoprotein cholesterol (LDL-C), calcium (Ca), and VB_2_ levels were significantly higher than those in the heat-stressed control group. Additionally, the amino acid composition of the yolk was improved. (4) Conclusion: CX enhances the nutrient content of egg yolks under both normal and high-temperature conditions, providing a valuable reference for the production of healthy and high-quality eggs.

## 1. Introduction

The Huaixiang chicken, a premium local breed in Guangdong Province, China, is renowned for its eggs’ exceptional nutritional value and distinctive flavor, garnering significant consumer preference. The yolk, a natural nutrient reservoir, represents the most concentrated source of bioactive components critical to human health. It provides all essential amino acids with high bioavailability, serving as an excellent source for muscle repair [1] and immune function support [2]. Notably, egg yolks are rich in triglycerides and lecithin, with lecithin enhancing lipid metabolism and improving gut health [3]. Triglycerides facilitate the absorption of fat-soluble vitamins (A, D, E, and K) in humans. Calcium and phosphorus, valuable mineral constituents in yolks, play pivotal roles: calcium is essential for skeletal mineralization, while phosphorus is crucial for ATP synthesis and energy metabolism. Additionally, egg yolks serve as a key dietary source of vitamin B_2_ (riboflavin), which promotes hepatic metabolism [4]. This comprehensive nutrient profile underscores the yolk’s multifunctional role in maintaining metabolic balance and physiological adaptability. Previous studies have demonstrated that nutritional interventions, such as supplementing laying hen diets with grape pomace, can enrich yolk phenolic content [5].

Canthaxanthin (CX), a carotenoid, exhibits significant potential as a feed additive for enhancing nutritional regulation in laying hens. Recent studies highlight its efficacy in improving yolk quality and optimizing production performance. Wen et al. reported that CX supplementation significantly increased the DSM score of fresh egg yolks through specific carotenoid deposition [6]. Independent studies by Maia et al. [7] and Zhao et al. [8] further confirmed that CX supplementation enhances egg production rate, egg weight, and relative yolk weight. Notably, Ismail demonstrated that adding 8 mg/kg CX to layer diets significantly increased yolk triglyceride content by 19.6% via modulation of lipid metabolism pathways [9]. These findings collectively suggest that dietary CX supplementation positively impacts poultry productivity and egg quality.

However, Huaixiang hens in Guangdong’s tropical/subtropical regions face prolonged heat stress under high-temperature and high-humidity conditions, which impairs lipid metabolism and consequently leads to significant adverse impacts on the nutritional quality of egg yolks [10]. Studies indicate that antioxidants (e.g., vitamin C and carotenoids) effectively mitigate heat-induced nutrient loss in yolks [11,12]. As a natural antioxidant, CX has been shown to alleviate oxidative stress under thermal challenges, thereby improving laying performance [13]. Nevertheless, research on CX’s role in enhancing Huaixiang hen yolk quality under heat stress remains scarce. Therefore, this study systematically evaluates the effects of dietary CX supplementation on egg production rate and yolk nutritional components (e.g., amino acids, lipids, and vitamin B2) in Huaixiang hens under thermoneutral and high-temperature conditions. The outcomes will provide valuable insights for the poultry industry to develop effective feeding strategies, enabling the production of healthier, premium-quality eggs that meet modern consumer demands.

## 2. Materials and Methods

### 2.1. Test Animals and Drugs

A total of 360, 25-week-old hens were purchased from Guangzhou Lilin Ecological Agriculture Co., Ltd. (Foshan, China). Canthaxanthin (CX, production batch number UE01611012) was purchased from DSM Vitamin Trading (Shanghai) Co., Ltd. (Shanghai, China),with a canthaxanthin content of 10%.

### 2.2. Feeding Management and Trial Design

In the laying hen breeding farm of Guangdong Ocean University, three layers of ladder cages were utilized. A total of 360 Huaixiang laying hens, with similar body weight and production performance at 25 weeks of age, were selected and randomly divided into 10 groups: 0 mg/kg, 4 mg/kg, 6 mg/kg, 8 mg/kg, and 10 mg/kg of CX food additive. The groups are detailed in Table 1, with 6 replicates per group and 6 laying hens per replicate. During the testing period, a combination of natural and artificial light was used, maintaining a light intensity of 10–15 Lux for 16 h (16 L:8 D). The environmental temperature and humidity were regulated using an insulation lamp and dehumidifier. The ambient temperature for the NC and NT groups was maintained at 25 ± 2 °C, while the temperature for the HC and HT groups was kept at 32 ± 2 °C from 9:00 to 17:00. Humidity for all groups was maintained at 65–75%. The temperature and relative humidity of the TN and HS groups were measured four times a day (Figure S1). The pre-test period lasted for 2 weeks (from 25 to 26 weeks), followed by a 9-week testing phase starting at 27 weeks (from 27 to 35 weeks). The corn–soybean meal-based diet was designed according to the Agricultural Industry Standard of the People’s Republic of China—Chicken Breeding Standard (NY/T 33-2004) [14] and local conditions, as shown in Table 2.

### 2.3. Sample Collection and Preservation

Egg collection was carried out on the final two days of the 3rd, 6th, and 9th weeks of the experiment. The eggs in each period were all stored in a refrigerator at 4 °C for no longer than 24 h. Two eggs were randomly sampled from each replicate sample for the measurement of egg weight, yolk weight, and yolk color. Moreover, one egg was randomly selected from each replicate sample for the detection of amino acid content, lipid content, vitamin B2 concentration, as well as the concentrations of calcium and phosphorus.

### 2.4. Determination Indices and Methods

#### 2.4.1. Measurement of Production Performance

The number of laying hens, feed intake, and egg production were recorded daily. The average daily feed intake was calculated using the following formula: Average daily feed intake = daily feed intake///number of laying hens. The daily egg production rate was calculated as follows: Daily egg yield = number of eggs produced///number of laying hens. The mean feed intake and egg production rates were calculated every 3 weeks based on the daily averages.

#### 2.4.2. Determination of Egg Yolk Weight and Color

The eggs were weighed, cracked, and separated to accurately measure the weight of the egg yolks. The relative weight of the yolk (%) was calculated as follows: Yolk weight (%) = (egg yolk weight (g)/egg weight (g)) × 100%. Yolk color was measured according to the DSM yolk color grading table (DSM Nutrition Products, Basel, Switzerland), ranging from 1 (light yellow) to 15 (dark orange) [15].

#### 2.4.3. Determination of Total Amino Acids in Egg Yolk

Approximately 0.5 g of the egg yolk sample was weighed (six replicate samples were configured for each group, and each sample was subjected to three measurements), and 5 mL of the extract was added at thermoneutral temperature. The mixture was centrifuged at 12,000 rpm at 4 °C for 10 min. The supernatant was placed on ice and analyzed using an amino acid determination kit (G0415W; Grace Biotechnology Co., Ltd., Suzhou, China) and a microplate reader (DNM-9606; Perlong Medical Co., Ltd., Beijing, China). The detection signals were measured at UV 570 nm.

#### 2.4.4. Determination of 17 Amino Acid Levels in Egg Yolk

The egg yolk sample was weighed (six replicate samples were configured for each group, and each sample was subjected to three measurements), and hydrochloric acid solution was added for homogenization. The acid hydrolysis method [16] was followed by PITC derivatization [17] and HPLC analysis (LC-100; Shanghai Wufeng Co., Ltd., Shanghai, China). The amino acids detected included aspartic acid, threonine, serine, glutamic acid, glycine, alanine, valine, methionine, isoleucine, leucine, tyrosine, phenylalanine, histidine, lysine, arginine, proline, and cysteine, adding up to a total of 17 amino acids. The detection signals were measured at UV 254 nm. The mobile phase A consisted of 0.05 mol/L sodium acetate (pH = 6.5), while the mobile phase B consisted of methanol (20%), acetonitrile (60%), and water (20%).

#### 2.4.5. Determination of Egg Yolk Lipid Levels

Weigh approximately 0.1 g of egg yolk samples (six replicate samples were configured for each group, and each sample was subjected to three measurements), add 1 mL of absolute ethanol, perform homogenization on ice, centrifuge at 12,000 rpm for 10 min at room temperature, collect the supernatant, and detect total cholesterol (TC, 510 nm, G0909W500; Grace Biotechnology, Suzhou, China), triglycerides (TG, 510 nm, G0910W; Grace Biotechnology, Suzhou, China), high-density lipoprotein cholesterol (HDL-C, 546 nm, G1221W; Grace Biotechnology, Suzhou, China), low-density lipoprotein cholesterol (LDL-C, 546 nm, G1222W; Grace Biotechnology, Suzhou, China), and lecithin (LEC, 510 nm, G1224W; Grace Biotechnology, Suzhou, China) using an enzyme colorimetric method with a commercial kit (Grace Biotechnology, Suzhou, China) and a microplate reader (DNM-9606; Perlong Medical Co., Ltd., Beijing, China).

#### 2.4.6. Determination of Vitamin B_2_ in Egg Yolk

Yolk samples were submitted to Suzhou Grus Biotechnology Co., Ltd. for analysis of vitamin B_2_ (VB_2_) (method 2.4.4.). The detection signals were measured at UV 522 nm. The mobile phase A consisted of 0.05 mol/L sodium acetate (pH = 6.5), while the mobile phase B consisted of methanol (65%) and water (35%).

#### 2.4.7. Determination of Egg Yolk Minerals

The calcium content within egg yolks was determined using a Thermo Fisher Scientific iCE 3500 atomic absorption spectrometer (Thermo Fisher Scientific, Massachusetts, USA). The sample was initially subjected to digestion treatment using 10 mL of nitric acid and 0.5 mL of perchloric acid. During the digestion process, it was maintained at a high temperature of 120 °C for 1 h, and then, the temperature was elevated to 180 °C for an additional 4 h. Thereafter, 20 g/L of Lanthanum standard solution was added to dilute the solution to 1 g/L. Ultimately, the absorbance value was determined at 422.7 nm and compared with the calcium carbonate standard solution for quantification.

A total of 4.5 g of the sample was accurately weighed; 80 mL of ether was added; and the mixture was allowed to soak for 24 h. After digestion with the perchloric acid–nitric acid digestion solution, it was reacted with ammonium vanadomolybdate. The phosphorus (P) content was determined via colorimetric measurement at 400 nm using a spectrophotometer (U-3900/3900H; Hitachi Limited, Tokyo, Japan).

### 2.5. Data Analysis

All test data were collected using Excel 2021 and analyzed statistically using IBM SPSS 26.0 software. The results were expressed as “mean ± standard deviation”. Data were analyzed using one-way analysis of variance (ANOVA). The Shapiro–Wilk test was used for normal distribution analysis (Table S1). Differences between the means were determined using Tukey’s multiple comparison post hoc test, with *p* < 0.05 considered statistically significant.

## 3. Results

### 3.1. Impact of CX Feeding at Normal Temperature on Egg-Laying Performance and Egg Yolk of Hens

#### 3.1.1. Impact of CX Feeding at Normal Temperature on Egg-Laying Performance

As shown in Table 3, the egg-laying rate exhibited a significant period-dependent increase across all groups (*p* < 0.05). In the 9-week test, the egg-laying rates of the NCX4, NCX6, and NCX8 groups were higher than that of the NC group (*p* < 0.05). Notably, dietary supplementation with 4 mg/kg CX resulted in the highest egg-laying rate, achieving a 31.0% increase relative to the NC group.

Similarly, the relative yolk weight increased progressively with hen ages. In the 3-week test, the relative yolk weight in the NCX4 and NCX6 groups was higher than that in the NC group (*p* < 0.05). Among the supplemented groups, 6 mg/kg CX yielded the most pronounced improvement, elevating yolk weight by 13.8% compared to the control.

#### 3.1.2. Impact of CX Feeding at Normal Temperature on Egg Yolk Color

**Figure 1:**
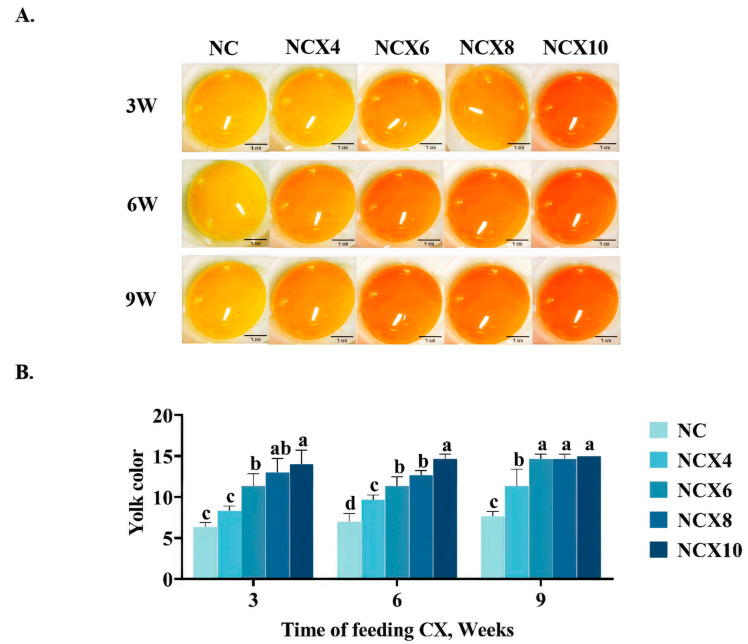
The effects of supplementing different concentrations of CX at normal temperature on DSM egg yolk color scores at 3, 6, and 9 weeks. (**A**) Representative images of egg yolks after continuous supplementation with different concentrations of CX for 3, 6, and 9 weeks under normal temperature; (**B**) The effect of continuous addition of CX for 3 weeks, 6 weeks, and 9 weeks on egg yolk color score under normal temperature. Scale bar 1 cm. Data for each treatment consist of 6 replicate samples, with each replicate comprising 2 eggs. The figure presents mean ± standard deviation. Different superscript letters ^a–d^ indicate significant differences (*p* < 0.05) among dietary CX supplementation doses within the same experimental period, as determined by one-way ANOVA. Abbreviations: NC, normal temperature control group; NCX4, supplementing the daily diet with an additional 4 mg/kg CX under normal temperature; NCX6, supplementing the daily diet with an additional 6 mg/kg CX under normal temperature; NCX8, supplementing the daily diet with an additional 8 mg/kg CX under normal temperature; NCX10, supplementing the daily diet with an additional 10 mg/kg CX under normal temperature.

During the three experimental periods, the color of the egg yolk improved significantly at normal temperatures (Figure 1). In the 3W trial, the yolk color scores in NCX6, NCX8, NCX10 were 21.5%, 28.7%, and 34.2% higher, respectively, compared to NC (*p* < 0.05). Similarly, in the 6W and 9W trials, the yolk color scores of the NCX4, NCX6, NCX8, and NCX10 groups also exhibited a significantly greater increase than in the NC group (*p* < 0.05) (Figure 1B). Notably, the 10 mg/kg CX supplementation consistently yielded the highest yolk color enhancement across all periods, with improvements of 121.2% (3W), 109.6% (6W), and 95.6% (9W) compared to baseline values.

#### 3.1.3. Impact of CX Feeding at Normal Temperature on Amino Acid Content of Egg Yolk

As shown in Table 4, the total amino acid content in the NCX6 and NCX8 groups during the 6W test was significantly lower than in the NC group (*p* < 0.05). However, during the 3W and 9W tests, no significant differences were observed among the NCX4, NCX6, NCX8, and NCX10 groups (*p* > 0.05), indicating that CX did not increase the total amino acid content at thermoneutral temperature. Additionally, the total amino acid content in the 3W and 6W yolk of the NC group was higher than that of the 9W group (*p* < 0.05).

As illustrated in Table 5, during the 3W trials, in the NCX10 group, the proportions of alanine, valine, and cysteine amino acids decreased. In contrast, in the NCX6 group, the proportion of Thr increased, whereas the proportion of cysteine decreased (*p* < 0.05). During the 6W trials, in the NCX6 group, the proportions of asparagine, lysine, glutamic acid, and threonine increased, whereas the proportions of cysteine decreased (*p* < 0.05). Notably, during the 9W trials, the effects of the NCX8 and NCX10 groups on amino acid profiles were consistent, leading to decreased proportions of leucine, histidine, alanine, valine, isoleucine, serine, and arginine while increasing the proportions of proline (*p* < 0.05).

#### 3.1.4. Impact of CX Feeding at Normal Temperature on Lipid Content of Egg Yolk

As shown in Table 6, dietary supplementation with graded CX doses (4–10 mg/kg) significantly elevated the LEC content in egg yolks compared to NC during both 6-week (*p* < 0.05) and 9-week trials (*p* < 0.05). Notably, the NCX10 group exhibited the highest LEC enrichment, with a 26.3% increase at 6 weeks, while the NCX6 group showed a 20.5% improvement at 9 weeks, highlighting dose- and time-specific efficacy.

Under the same dosing conditions, the LEC contents of the NC, NCX4, NCX6, NCX8, and NCX10 in the 6W and 9W groups were higher than those in the 3W group (*p* < 0.05), indicating that the LEC deposition in the 6W and 9W groups was greater than that in the 3W group, and the addition of CX did not alter this pattern.

#### 3.1.5. Impact of CX Feeding at Normal Temperature on Other Nutrients of Egg Yolk

As indicated in Table 7, under normal temperature conditions, supplementation with 4–10 mg/kg CX did not significantly affect the deposition of Ca, P, and VB_2_ in egg yolks within the same time period (*p* > 0.05). Notably, the VB_2_ content in the NCX10 group was significantly higher at 6 weeks compared to 3 and 9 weeks, indicating that VB_2_ levels peaked at 6 weeks and subsequently declined. This suggests that the enrichment effect of CX on VB_2_ may diminish with prolonged feeding duration.

### 3.2. Impact of CX Feeding at High Temperature on Egg-Laying Performance and Egg Yolk of Hens

#### 3.2.1. Impact of CX Feeding at High Temperature on Egg-Laying Performance

##### Effect of High Temperature on Egg-Laying Performance

As shown in Table 8, the egg-laying rate in the experimental 6W and 9W HC groups was significantly lower than that in the NC group (*p* < 0.05), indicating that prolonged exposure to high temperatures negatively affected the laying rate compared to normal temperature. Additionally, the yolk weight in the 6W HC groups was lower than that in the NC group (*p* < 0.05). Moreover, the relative yolk weight of the HC group was lower than that of the NC group at 9W (*p* < 0.05). These results collectively demonstrate that sustained heat stress disrupts both egg-laying efficiency and yolk partitioning in laying hens.

##### Impact of CX Feeding at High Temperature on Egg-Laying Performance

As shown in Table 8, the egg production in the 3W HCX6 and HCX8 groups was significantly higher than in the HC group (*p* < 0.05). Similarly, in the 6W HCX6, HCX8, and HCX10 groups, the production rates were higher than those in the HC group (*p* < 0.05). In the 9W trial, egg production in the HCX4, HCX6, HCX8, and HCX10 groups also exceeded that of the HC group (*p* < 0.05). Among the three experimental periods, the addition of 8 mg/kg CX improved the laying rates by 30%, 67.7%, and 69.7%, respectively. During these periods, no significant differences in the laying rate were observed between the HCX8 group and the NC group (*p* > 0.05), indicating that the addition of 8 mg/kg CX at high temperatures returned the laying rates to normal levels, with rates at 3W, 6W, and 9W being 108%, 92.9%, and 96.6% of normal temperature rates, respectively.

As shown in Table 8, in the 3W trial, yolk weights in the HCX8 groups were higher than in the NC group (*p* < 0.05), with the addition of 8 mg/kg CX improving yolk weight by 19.5%. In the 6W trial, yolk weights in the HCX6, HCX8, and HCX10 groups exceeded those in the HC group (*p* < 0.05), and yolk weight increased by 12.6% with 10 mg/kg CX, with no significant difference compared with the NC group (*p* > 0.05). In the 9W trial, yolk weight in the HCX8 group was significantly greater than in the HC group (*p* < 0.05), with an increase of 20.5%, showing no significant difference compared with the NC group. These findings indicate that adding CX at 3W can elevate yolk weight above normal levels, while 10 mg/kg CX at 6W and 8 mg/kg CX at 9W can restore yolk weight to normal levels, achieving 97.9% and 109.3% of the normal temperature weight, respectively.

As shown in Table 8, in the 3W trial, the relative weights of egg yolk in the HCX4 and HCX8 groups were higher than in the NC group (*p* < 0.05). In the 9W trial, the relative weight of egg yolk in the HCX8 group was higher than in the HC group (*p* < 0.05) and was not significantly different from the NC group. These results indicate that under high-temperature conditions, dietary supplementation with CX for 3 weeks increases the relative weight of egg yolks above normal levels; however, continuous supplementation for 6 weeks has no significant effect on egg yolk relative weight; by 9 weeks, continuous supplementation restores the relative weight of egg yolks to normal levels.

#### 3.2.2. Impact of CX Feeding at High Temperature on Egg Yolk Color

**Figure 2:**
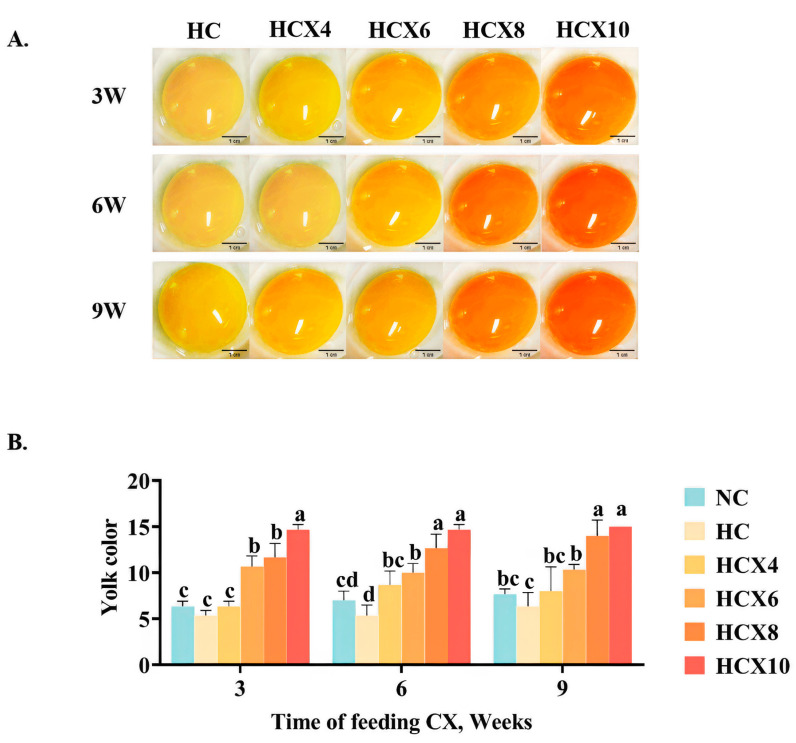
The effects of continuous high temperatures for 3, 6, and 9 weeks on DSM egg yolk color scores, as well as the effects of continuous supplementation with varying concentrations of CX for 3, 6, and 9 weeks under high temperature on DSM egg yolk color scores, were evaluated. (**A**) Representative images of egg yolks after continuous supplementation with different concentrations of CX for 3, 6, and 9 weeks under high temperature; (**B**) The effect of continuous addition of CX for 3 weeks, 6 weeks, and 9 weeks on egg yolk color score under high temperature. Scale bar 1 cm. Data for each treatment consist of 6 replicate samples, with each replicate comprising 2 eggs. The figure presents mean ± standard deviation. Different superscript letters ^a–d^ indicate significant differences (*p* < 0.05) among dietary CX supplementation doses within the same experimental period, as determined by one-way ANOVA. Abbreviations: NC, normal temperature control group; HC, high-temperature control group; HCX4, supplementing the daily diet with an additional 4 mg/kg CX under high temperature; HCX6, supplementing the daily diet with an additional 6 mg/kg CX under high temperature; HCX8, supplementing the daily diet with an additional 8 mg/kg CX under high temperature; HCX10, supplementing the daily diet with an additional 10 mg/kg CX under high temperature.

Figure 2B indicates that there was no significant difference in yolk color scores between the HC and NC groups during the three testing periods (*p* > 0.05).

Figure 2B shows that, compared to the HC group, the yolk color scores significantly increased in the HCX6, HCX8, and HCX10 groups during the 3W and 9W trials (*p* < 0.05). In the 6W trial, the yolk color scores of the HCX4, HCX6, HCX8, and HCX10 groups also exhibited significant increases (*p* < 0.05). The addition of 10 mg/kg CX at high temperatures resulted in yolk color score increases of 175.2%, 175.2%, and 137.0% for the 3W, 6W, and 9W periods, respectively.

Compared to the NC group, the yolk color scores significantly increased in the HCX6, HCX8, and HCX10 groups during the 3W and 6W trials (*p* < 0.05). In the 9W trial, significant yolk color score increases were observed in the HT3 and HT4 groups (*p* < 0.05). The addition of 10 mg/kg CX at high temperatures improved yolk color across all periods, with increases of 237.6%, 209.6%, and 195.6% at normal temperatures for the 3W, 6W, and 9W trials, respectively.

#### 3.2.3. Impact of CX Feeding at High Temperature on Amino Acid Content of Egg Yolk

In the 3W and 6W trials, the total amino acid content in the HC group was significantly lower than in the NC group (*p* < 0.05) (Table 9). As shown in Table 10, in the 3W trial, the arginine level in the HC group was significantly higher than that in the NC group (*p* < 0.05), whereas the cysteine level in the HC group was significantly lower than that in the NC group (*p* < 0.05). Additionally, in the 6W trials, compared with the NC group, the proportion of valine in the HC group was significantly increased, whereas the proportion of cysteine was significantly decreased (*p* < 0.05).

The results presented in Table 9 indicate that after 3 weeks of the experiment, the total amino acid content in the HCX4, HCX6, and HCX8 groups did not differ significantly from that in the HC group but was significantly lower than that in the NC group (*p* > 0.05). Similarly, after 6 weeks, the total amino acid content in the HCX4, HCX6, HCX8, and HCX10 groups also did not differ significantly from that in the HC group but remained significantly lower than that in the NC group (*p* > 0.05). In the 3W trials, compared with the HC group, the proportions of valine, isoleucine, and arginine decreased, while proline increased in the HCX6 and HCX8 groups (*p* < 0.05) (Table 10). In the 6W trials, the proportion of valine in the HCX6, HCX8, and HCX10 groups was significantly lower than that in the HC group (*p* > 0.05). However, in the 9W trials, lysine, glutamic acid, and arginine proportions in the HCX10 group were significantly lower than in the HC group (*p* < 0.05).

#### 3.2.4. Impact of CX Feeding at High Temperature on Lipid Content of Egg Yolk

##### Effect of High Temperature on Egg Yolk Lipid Content

As shown in Table 11, the test at 9W indicated that the HC group had increased TC content compared to the NC group (*p* < 0.05). As indicated in Table 11, the 9W test showed a decrease in LEC content in the HC group compared to the NC group (*p* < 0.05).

##### Impact of CX Feeding at High Temperature on the Lipid Content of Egg Yolk

- As shown in Table 11, the results of the 3W experiment showed that the TG content in the HCX6, HCX8, and HCX10 groups was significantly increased compared with the NC group (*p* < 0.05). This indicates that continuous addition of CX in a high-temperature environment can improve yolk TG levels to beyond those observed at normal temperature, with an optimal dose of 10 mg/kg CX leading to a 121.4% increase in yolk TG content.
- As presented in Table 11, compared with the HC group, the 3W test in the HCX8 group led to an increase (*p* < 0.05), with the addition of 8 mg/kg CX resulting in a 22.9% increase in yolk TC content. Furthermore, compared with the NC group, the HCX4, HCX8, and HCX10 groups exhibited significant increases in TC content during the 3W experiment (*p* < 0.05). Notably, the addition of 8 mg/kg CX elevated the TC levels to 133.2% of those observed under normal temperature conditions. In the 9W test, the HCX6 and HCX10 groups also showed an increase (*p* < 0.05), with 10 mg/kg CX raising the TC content to 118.0% of that observed under normal temperature.
- As shown in Table 11, compared with the HC group, in the 6W test, the HCX4, HCX6, HCX8, and HCX10 groups exhibited significant increases (*p* < 0.05), with the addition of 6 mg/kg CX leading to a 25.8% rise in yolk LEC content. Similarly, compared with the HC group, in the 9W test, the HCX4, HCX6, HCX8, and HCX10 groups also demonstrated an increase (*p* < 0.05), with 4 mg/kg CX improving the yolk LEC content by 26.7%. In the 6W test, the addition of 6 mg/kg CX resulted in yolk LEC content reaching 122.7% of normal temperature levels (*p* < 0.05). In the 9W test, the yolk LEC content similarly increased to 114.7% of normal temperature levels with the addition of 4 mg/kg CX (*p* < 0.05).

As presented in Table 11, following continuous supplementation of CX for 9 weeks, the LDL-C content in the HCX8 group was significantly lower than that in the HC group (*p* < 0.05), whereas the LDL-C content in the HCX4 group was significantly higher than that in the NC group (*p* < 0.05).

As presented in Table 11, compared with the NC group, HDL-C content in the HCX10 group increased in both 6W and 9W trials (*p* < 0.05), with increases of 41.8% and 25.8%, respectively. Additionally, compared with the HC group, HDL-C content in the HCX10 group showed a significant increase between 6W and 9W trials (*p* < 0.05), with increases of 49.8% at 6W and 25.1% at 9W.

#### 3.2.5. Impact of CX Feeding at High Temperature on Other Nutrients of Egg Yolk

Table 12 indicates that the 3W test showed lower yolk Vb2 levels in the HC group than in the NC group (*p* < 0.05), suggesting that high temperature inhibited yolk Vb2 in the yolk during the early stage of the trial. Additionally, the Vb2 content in the HC group at 3W was significantly higher than that at 6W and 9W (*p* < 0.05), indicating that prolonged exposure to high temperature resulted in a greater reduction in yolk Vb2 content compared to the HC group.

In the 3W test, the yolk Ca levels in the HCX6, HCX8, and HCX10 groups increased compared with the HC group (*p* < 0.05), and the yolk Ca content increased by 15.1% after adding 10 mg/kg CX. In the 9W test, the HCX6 and HCX10 groups also showed significant increases compared with the HC group (*p* < 0.05), and the addition of 10 mg/kg CX led to a 30.2% rise in yolk Ca content (Table 12). In the 3W, 6W, and 9W trials, adding 10 mg/kg, 8 mg/kg, and 10 mg/kg, respectively, increased the calcium levels in egg yolks by 22.0%, 25.6%, and 23.2% compared with the NC group.

Compared with the HC group, the yolk Vb2 content in the HCX6, HCX8, and HCX10 groups significantly increased in the 6W test (*p* < 0.05), and the VB_2_ content increased by 128.5% after adding 6 mg/kg CX. In the 9W test, compared with the HC group, the VB_2_ levels in the HCX6 and HCX10 groups were significantly elevated (*p* < 0.05). Following the addition of 6 mg/kg CX, the VB_2_ content in the egg yolk increased by 237.0% (Table 12).

## 4. Discussion

### 4.1. The Impact of CX on the Nutritional Composition of Egg Yolk at Normal Temperature

The results of this study indicate that continuous supplementation with CX for 9 weeks at a normal temperature significantly enhances the laying rate. Additionally, a 3-week continuous addition of CX increases the relative weight of the egg yolk. Several earlier studies have confirmed that CX can enhance egg yield, egg weight, and yolk weight in laying hens [18,19]. However, in our experiment, feeding CX at normal temperature did not result in a significant increase in egg weight or yolk weight. The inconsistent experimental results may be attributed to variations in hen breeds. Based on our findings, we speculate that CX influences the quality of egg yolks and albumen by modulating the allocation of nutrients during the egg formation process. This is supported by previous studies demonstrating that CX promotes ovarian follicle development in hens [8], leading to preferential protein allocation to yolk formation during the reproductive period. Consequently, this may result in relatively lower albumen quality and ultimately contribute to an increase in the relative weight of the yolk.

The color depth of egg yolk is often used as an indicator of the nutritional value of eggs. Therefore, improving yolk color in production is essential. In this study, it was observed that higher concentrations of CX significantly enhanced the DSM color score of egg yolks at thermoneutral temperature, consistent with previous findings [6,18]. This dose-dependent effect can be mechanistically attributed to CX’s structural properties: its nine conjugated double bonds and dual oxygen substituents at positions 4 and 4′ of the β-ionone ketone backbone confer distinct orange pigmentation and high lipid solubility [20]. Upon dietary supplementation with CX, it rapidly dissolves into free fatty acids and is absorbed by intestinal epithelial cells. Subsequently, CX is transported to the liver, where it is incorporated into lipoproteins. These lipoproteins are then transported via the bloodstream to the ovary, ultimately depositing in the developing yolk [21]. This pathway explains the positive correlation between CX supplementation levels and elevated DSM chroma scores observed in our experiments.

This study revealed that total amino acid content in egg yolks decreased with advancing hen age, a trend that remained unaltered by CX supplementation. Consistent with findings reported by Santos et al. [22], this phenomenon may stem from the shift in nutrient allocation during the late peak laying period, where resources are redirected from egg production to self-maintenance—a metabolic priority that CX failed to counteract. Numerous studies have demonstrated the capacity of feed additives to modify yolk amino acid profiles [23,24], and our results further corroborate this premise. Specifically, CX supplementation reduced the proportions of cysteine, alanine, valine, lysine, leucine, histidine, isoleucine, and arginine in egg yolks, the amino acids collectively associated with bitter taste perception [25,26]. This result suggests that CX may effectively mitigate the bitterness of egg yolks by reducing the proportion of amino acids associated with bitter taste perception, thereby enhancing the overall flavor profile of the egg yolks. Notably, prolonged administration of 6 mg/kg CX for 3 and 6 weeks significantly elevated threonine levels in yolks. As an essential amino acid for humans [27], threonine plays pivotal roles in cellular proliferation regulation, immune response modulation, and critical brain development processes [28]. Furthermore, the supplementation of CX significantly increased the proline content in the egg yolk, which is a crucial building block for the synthesis of elastin, a key structural protein crucial for the integrity and mechanical strength of connective tissues like bones, skin, cartilage, and vascular systems [29].

Almost all lipids in eggs are concentrated in the yolk, comprising about 65% of the dry matter, with 62% as triglycerides (TG), 33% as phospholipids, and cholesterol (TC) constituting less than 5% [30]. The triglycerides in the yolk serve multiple functions in the body, participate in metabolism, and act as the primary energy source for the chicken embryo. The composition of yolk phospholipids is unique compared to other sources, such as soybean phospholipids, where lecithin (LEC) is the main component (66%) [31]. Lecithin is widely utilized in cosmetics, pharmaceuticals, and feed production [32]. More than 95% of the cholesterol in the yolk is free cholesterol, and the interactions between adjacent phospholipids are crucial for maintaining the lipoprotein structure [33]. At normal temperatures, adding CX can increase the LEC content of egg yolk. Despite the limited number of studies investigating the impact of CX on enhancing egg yolk lipid content, Panaite found that feeding carotenoids can increase the cholesterol levels in egg yolk [34]. This effect may be attributed to CX enhancing estrogen secretion, promoting VLDL synthesis, which are key factors influencing the lipid content of egg yolks.

Trace elements in eggs are vital for supplementing human nutritional needs. Since the human body cannot synthesize Ca, P, or Vb2, these nutrients must be obtained through diet, making eggs an important source. Therefore, this study examined the impact of dietary CX supplementation on the concentrations of Ca, P, and Vb2 in egg yolks. The results demonstrated that dietary CX supplementation had no significant effect on the deposition of Ca, P, and Vb2 in egg yolks. To date, no studies have investigated the impact of CX on the deposition of Ca, P, and Vb2 in egg yolks. However, research by Douglas et al. demonstrated that CX had no significant effect on the calcium and phosphorus concentrations in chicken tibias [35].

### 4.2. The Impact of CX on the Nutritional Composition of Egg Yolk at High Temperature

In this study, heat stress resulted in decreased laying rates, yolk weight, and overall egg weight. Kim et al. and Barrett et al. also confirmed that heat stress negatively impacts production performance, including feed intake and egg production rates [36,37]. This decline can be attributed to the inhibition of feed intake via the hypothalamic–pituitary–adrenal (HPA) axis in a high-temperature environment, which reduces the heat generated by digestion and absorption, thereby limiting the energy available for egg production. Furthermore, the inhibition of estradiol synthesis may also contribute to the reduction in yolk weight and specific gravity. Heat stress increases the cellular levels of heat shock protein 70 (HSP70), which downregulates the activity of the follicle-stimulating hormone receptor (FSHR) and cytochrome P450 19A1 (CYP19A1) promoters, leading to impaired estradiol synthesis [38]. In the liver, estradiol binds to estrogen receptors to promote the synthesis of vitellogenin (VTG) and very-low-density lipoprotein (VLDL), both critical components of the yolk [39,40,41]. Meanwhile, the downregulation of cytochrome P450 11A1 (Cyp11a1) and steroidogenic acute regulatory protein (STAR) further impairs progesterone synthesis [42], contributing to the observed decrease in laying rates. In the high-temperature environment of this experiment, feeding CX increased the egg production rate throughout the study period. The findings are in agreement with those reported by Sun et al. [13]. It is likely that long-term CX supplementation enhances the antioxidant capacity of serum and ovarian tissue [43], thereby mitigating oxidative-stress-induced apoptosis in granulosa cells, reducing the number of atretic follicles, promoting the secretion of reproductive hormones and the expression of their receptors, facilitating follicular development and maturation, and ultimately increasing the egg production rate [13]. Furthermore, the results of this study demonstrate that during the initial three-week period, CX significantly increased the relative weight of the egg yolk. To date, no specific studies have explored the mechanism underlying the effect of CX on yolk relative weight under high-temperature conditions. We speculate that this effect may be attributed to CX enhancing the expression of estradiol [44], FSHR, and Cyp11a1 in granulosa cells [45], ultimately resulting in the observed increase in yolk relative weight.

In this study, high temperature did not affect yolk color, which is consistent with the findings of Wang et al. [46]. However, Kim et al. [36] reported that heat stress (33 °C, 66% RH) led to a reduction in egg yolk color. The underlying cause of this discrepancy remains unclear; we speculate that it may be attributed to differences in hen breeds. In our experiment, higher CX supplementation under high-temperature conditions resulted in elevated DSM chroma scores. We hypothesize that this effect follows the same mechanism observed under thermoneutral conditions.

Our findings indicate that the total amino acid content in egg yolks decreases under high-temperature conditions, consistent with the observations reported by Chen [10]. Additionally, our findings indicate that high-temperature exposure decreases the proportion of cysteine in egg yolks. However, the proportion of arginine in egg yolks increases after 3 weeks of continuous high temperature, and the proportion of valine in egg yolks rises after 6 weeks of sustained high-temperature conditions. We hypothesize that heat stress disrupts the amino acid balance in hens, leading to increased demands for amino acid and energy supplementation in laying hens. This results in a reduced total amino acid concentration in egg yolks under heat stress conditions, as well as adjustments in the proportions of cysteine, arginine, and valine [47]. Furthermore, our study demonstrates that under high-temperature conditions, dietary supplementation with CX increases the proportion of proline while concurrently decreasing the proportions of isoleucine, valine, arginine, lysine, and glutamic acid. Proline in egg yolk is associated with sweetness and caramel flavor [48], whereas isoleucine, valine, arginine, and lysine are linked to bitterness and fishy odor [49]. The addition of CX under high-temperature conditions can modulate amino acid metabolism, thereby balancing the bitter and sweet profiles of egg yolk, suppressing off-flavors, enhancing flavor complexity, and ultimately optimizing overall sensory quality.

This study demonstrated that high temperatures reduced yolk TG and LEC content, aligning with the findings of Akdemir [50]. The likely mechanism is that TG in hepatocytes are primarily transported to the yolk via very-low-density lipoprotein (VLDL) [51], while LEC is mainly transported to the yolk as vitellogenin (VTG) [52]. Heat-stress-induced damage to granulosa cells leads to reduced estrogen secretion [38], which in turn inhibits hepatic synthesis of VLDL and VTG [40]. This ultimately results in decreased TG and LEC content in egg yolks. Additionally, the increase in yolk TC content at elevated temperatures later in the test was consistent with Otaibi’s study [53]. This result can be explained by the action of glucocorticoids, which are released because of the hypothalamic–pituitary–adrenal axis stimulation in HS birds [54]. The findings of this study indicate that high-temperature feeding with CX significantly increases the levels of TG, TC, LEC, and HDL-C in egg yolks. Notably, the levels of TG and LEC surpass those observed in the control group fed at normal temperatures, highlighting the substantial impact of CX in promoting lipid deposition in egg yolks. The TG in egg yolks serve not only as efficient energy carriers but also as critical mediators for essential fatty acids, fat-soluble vitamins, and the regulation of cellular functions. A moderate intake of egg yolk TG, when integrated with a balanced diet, can contribute to optimizing metabolic health and reducing the risk of chronic diseases [55]. TC is a critical component of cellular structure and plays an essential role in the synthesis of hormones and vitamins in the human body. Traditionally, it has been believed that dietary cholesterol, such as that found in egg yolks, can elevate blood cholesterol levels. However, recent research indicates that TC in egg yolks is often associated with choline and lutein, which may enhance lipid metabolism and cognitive function [56]. The cumulative benefits of these compounds may surpass the potential risks associated with dietary cholesterol alone. LEC in egg yolks serves as a multifunctional nutritional carrier, playing a critical role in various physiological processes, from cell structure development to the prevention of chronic diseases. In conclusion, supplementing the diets of hens in high-temperature environments with CX can significantly increase the lipid content of egg yolks, thereby enhancing the overall nutritional quality of eggs under these conditions.

This study found that high temperatures decreased the VB_2_ content in egg yolk. While there are no previous studies specifically addressing the impact of high temperature on yolk VB_2_, Deyhim et al. reported a 37% reduction in VB_2_ in thoracic muscle due to heat stress without additional vitamin supplementation [57]. The liver synthesizes riboflavin-binding protein (RBP) [58], which binds to VB_2_ during its release into the bloodstream, forming the RBP–VB_2_ complex. This complex subsequently binds to VTG dispersed in the blood, facilitating transport to the follicle [59]. Both RBP and VTG are synthesized in the liver under the influence of estrogen [60,61]. Therefore, we speculate that heat stress may impair estrogen secretion, thereby affecting the transport of VB_2_ to the yolk. This study demonstrates that under high-temperature conditions, dietary supplementation with CX can significantly enhance the levels of calcium and VB_2_ in eggs. However, the underlying molecular mechanisms, such as whether CX modulates the expression of calcium-binding proteins or riboflavin transporters, require further investigation.

## 5. Conclusions

Overall, under normal temperature conditions, dietary supplementation with CX significantly enhances egg yolk quality. In high-temperature environments, CX addition effectively mitigates the adverse effects of heat stress on egg yolk quality, thereby offering potential health benefits to consumers. 

## Figures and Tables

**Table 1:** Arrangement of the treatments.

Group	NC	NCX4	NCX6	NCX8	NCX10	HC	HCX4	HCX6	HCX8	HCX10
Temperature	Normal temperature (25 ± 2 °C)					High temperature (32 ± 2 °C)				
Supplementary dose of CX (mg/kg)	0	4	6	8	10	0	4	6	8	10

**Table 2:** Composition and nutrient levels of basal diets (air-dry basis).

Ingredients	Percent (%)	Nutrients ^2^ (Analyzed Composition, %)	Content (%)
Corn	55.0	ME/(MJ/kg)	11.3
Wheat bran	9.5	Ca, %	3.0
Soybean meal	20.0	CP, %	15.5
Fish meal	5.0	TP, %	0.6
Limestone	7.5	Met, %	0.4
Ca(HCO_3_)_2_	2.5	Cys, %	0.3
NaCl	0.1	Lys, %	0.8
Premix ^1^	0.4		

Note: ^1^ The premix provided the following per kilogram of diet: vitamin A 9000 IU, vitamin D 2500 IU, vitamin E 20 IU, vitamin B12 12 µg, vitamin K 2.4 mg; Microelements: Mn 100 mg, Zn 60 mg, Fe 25 mg, Cu 5 mg, Co 0.1 mg (sulfates form); Se(Na_2_SeO_3_) 0.2 mg, I (KI) 0.5 mg. ^2^ Except for metabolic energy (ME), others are measured values.

**Table 3:** Effects of dietary CX supplementation under normal temperature on egg-laying performance in hens: a comprehensive analysis of egg-laying rate, average egg weight, average feed intake, yolk weight, and relative weight of egg yolk over 3-week, 6-week, and 9-week periods. Data are presented as mean ± standard deviation. *n* = 6 replicates (6 hens per replicate).

Item	Time	NC	NCX4	NCX6	NCX8	NCX10	*p*-Value
Egg-laying rate (%)	3 weeks	47.61 ± 5.61 ^B^	39.64 ± 3.75 ^B^	39.86 ± 1.87 ^B^	45.08 ± 2.14 ^B^	39.17 ± 1.24 ^AB^	0.323
	6 weeks	55.63 ± 8.18 ^B^	43.27 ± 5.06 ^B^	40.55 ± 8.32 ^B^	44.33 ± 3.99 ^B^	34.26 ± 7.21 ^B^	0.328
	9 weeks	67.80 ± 3.81 ^Ab^	76.96 ± 2.55 ^Aa^	73.45 ± 2.02 ^Aa^	75.18 ± 3.45 ^Aa^	54.91 ± 0.41 ^Ac^	0.000
	*p*-value	0.046	0.001	0.005	0.00	0.042	
Average egg weight (g)	3 weeks	42.93 ± 1.22	40.6 ± 1.6	40.69 ± 0.72 ^B^	41.35 ± 0.94	41.1 ± 0.79	0.577
	6 weeks	42.66 ± 1.17	41.08 ± 3.26	45.39 ± 0.73 ^A^	43.58 ± 1.94	42.79 ± 1.36	0.614
	9 weeks	42.14 ± 1.97	45.61 ± 2.22	46.71 ± 1.06 ^A^	41.14 ± 1.61	44.04 ± 1.22	0.138
	*p*-value	0.930	0.311	0.000	0.486	0.225	
Average feed intake (g)	3 weeks	110.72 ± 18.62	97.12 ± 21.54	97.89 ± 21.63	98.52 ± 21.56	93.15 ± 25.17	0.983
	6 weeks	102.9 ± 7.42	91.82 ± 10.97	98.89 ± 8.22	92.16 ± 6.17	84.08 ± 5.7	0.533
	9 weeks	101.42 ± 8.61	88.4 ± 4.24	94.16 ± 6.42	93.01 ± 4.36	89.2 ± 5.56	0.589
	*p*-value	0.8578	0.910	0.968	0.934	0.916	
Yolk weight (g)	3 weeks	12.44 ± 0.57 ^B^	13.2 ± 0.31 ^B^	13.52 ± 0.37 ^B^	12.41 ± 0.28 ^B^	12.92 ± 0.44 ^B^	0.263
	6 weeks	15.94 ± 0.76 ^A^	15.04 ± 0.52 ^A^	16.83 ± 0.54 ^A^	15.68 ± 0.47 ^A^	15.54 ± 0.69 ^A^	0.341
	9 weeks	14.82 ± 0.71 ^AB^	15.48 ± 0.62 ^A^	16.15 ± 0.3 ^A^	14.95 ± 0.46 ^A^	15.22 ± 0.61 ^A^	0.483
	*p*-value	0.008	0.013	0.00	0.00	0.013	
Relative weight of egg yolk (%)	3 weeks	29.1 ± 1.65 ^Bb^	32.69 ± 1.12 ^a^	33.22 ± 0.62 ^Ba^	30.11 ± 1.06 ^Bab^	31.41 ± 0.73 ^Bab^	0.039
	6 weeks	35.49 ± 1.95 ^A^	34.57 ± 2.52	34.17 ± 1.6 ^AB^	34.34 ± 1.92 ^A^	34.47 ± 1.79 ^AB^	0.987
	9 weeks	37.26 ± 1.26 ^A^	37.53 ± 1.57	37.61 ± 0.51 ^A^	36.49 ± 1.23 ^A^	37.52 ± 0.78 ^A^	0.516
	*p*-value	0.007	0.154	0.047	0.01	0.028	

Note: ^A,B^ Values within a column with different superscripts differ significantly at *p* < 0.05, while those with the same or no letter superscripts mean no significant difference (*p* > 0.05). ^a–c^ Values within a row with different superscripts differ significantly at *p* < 0.05, while those with the same or no letter superscripts mean no significant difference (*p* > 0.05). Abbreviations: NC, normal temperature control group; NCX4, supplementing the daily diet with an additional 4 mg/kg CX under normal temperature; NCX6, supplementing the daily diet with an additional 6 mg/kg CX under normal temperature; NCX8, supplementing the daily diet with an additional 8 mg/kg CX under normal temperature; NCX10, supplementing the daily diet with an additional 10 mg/kg CX under normal temperature.

**Table 4:** Effects of dietary CX supplementation under normal temperature on total amino acid content in egg yolks at 3, 6, and 9 weeks. Data are presented as mean ± standard deviation. *n* = 6 replicates.

Total Amino Acid Content (μmol/g)	3 Weeks	6 Weeks	9 Weeks	*p*-Value
NC	123.99 ± 1.95 ^AB^	137.24 ± 4.42 ^Ab^	110.75 ± 9.81 ^B^	0.012
NCX4	117.36 ± 2.64	123.26 ± 2.87 ^ab^	111.46 ± 9.40	0.153
NCX6	113.42 ± 8.92	106.01 ± 7.64 ^bc^	120.85 ± 4.60	0.245
NCX8	116.50 ± 4.20 ^AB^	108.68 ± 2.74 ^Bc^	124.31 ± 3.99 ^A^	0.013
NCX10	124.37 ± 4.28	128.92 ± 6.53 ^ab^	119.83 ± 5.76	0.41
*p*-value	0.086	0.008	0.151	

Note: ^A,B^ Values within a column with different superscripts differ significantly at *p* < 0.05, while those with the same or no letter superscripts mean no significant difference (*p* > 0.05). ^a–c^ Values within a row with different superscripts differ significantly at *p* < 0.05, while those with the same or no letter superscripts mean no significant difference (*p* > 0.05). Abbreviations: NC, normal temperature control group; NCX4, supplementing the daily diet with an additional 4 mg/kg CX under normal temperature; NCX6, supplementing the daily diet with an additional 6 mg/kg CX under normal temperature; NCX8, supplementing the daily diet with an additional 8 mg/kg CX under normal temperature; NCX10, supplementing the daily diet with an additional 10 mg/kg CX under normal temperature.

**Table 5:** Effects of dietary CX supplementation under normal temperature on percentages of 17 individual amino acids in egg yolks at 3, 6, and 9 weeks. Data are presented as mean ± standard deviation. *n* = 6 replicates.

Proportion (%)	Time	NC	NT1	NT2	NT3	NT4	*p*-Value
Leucine	3 weeks	7.51 ± 0.12	7.54 ± 0.23	7.88 ± 0.43	7.65 ± 0.27	7.19 ± 0.12	0.489
	6 weeks	7.13 ± 0.35	7.36 ± 0.19	8.79 ± 0.68	8.74 ± 0.51	7.44 ± 0.33	0.056
	9 weeks	8.04 ± 0.31 ^a^	7.75 ± 0.37 ^ab^	7.1 ± 0.29 ^bc^	6.7 ± 0.25 ^c^	6.94 ± 0.16 ^bc^	0.035
Asparagine	3 weeks	4.4 ± 0.02	4.96 ± 0.71	5.41 ± 0.09	4.68 ± 0.01	4.58 ± 0.01	0.126
	6 weeks	3.33 ± 0.13 ^b^	4.71 ± 0.57 ^a^	5.07 ± 0.05 ^a^	4.24 ± 0.35 ^ab^	3.53 ± 0.23 ^b^	0.008
	9 weeks	5.78 ± 0.13	5.23 ± 0.83	5.71 ± 0.13	5.06 ± 0.28	5.72 ± 0.29	0.437
Histidine	3 weeks	1.5 ± 0.02	1.49 ± 0.1	1.53 ± 0.01	1.48 ± 0.08	1.34 ± 0.03	0.198
	6 weeks	1.59 ± 0	1.62 ± 0.03	1.9 ± 0.07	1.88 ± 0.15	1.59 ± 0.01	0.112
	9 weeks	1.4 ± 0.04 ^a^	1.36 ± 0.17 ^ab^	1.21 ± 0.04 ^ac^	1.14 ± 0.01 ^bc^	1.09 ± 0.06 ^c^	0.027
Lysine	3 weeks	6.07 ± 0.03	6.11 ± 0.53	6.78 ± 0.08	6.04 ± 0.02	5.55 ± 0.24	0.086
	6 weeks	5.5 ± 0.08 ^b^	6.44 ± 0.25 a^b^	7.52 ± 0 ^a^	6.8 ± 0.2 ^ab^	5.58 ± 0.26 ^b^	0.027
	9 weeks	6.86 ± 0.17	5.7 ± 0.92	6.16 ± 0.15	5.38 ± 0.14	5.54 ± 0.22	0.094
Glutamic acid	3 weeks	4.38 ± 0.26	5.08 ± 0.77	5.59 ± 0.06	4.79 ± 0.04	4.6 ± 0.12	0.135
	6 weeks	4.34 ± 0.46 ^b^	5.31 ± 0.02 ^ab^	6.23 ± 0.14 ^a^	5.43 ± 0.58 ^ab^	4.47 ± 0.51 ^b^	0.026
	9 weeks	4.47 ± 0.02	4.06 ± 1.08	5.05 ± 0	4.22 ± 0.42	4.73 ± 0.32	0.474
Alanine	3 weeks	3.18 ± 0.1 ^ab^	3.26 ± 0.03 ^a^	3.02 ± 0.09 ^ab^	2.88 ± 0.05 ^bc^	2.55 ± 0.2 ^c^	0.008
	6 weeks	3.33 ± 0.1	3.44 ± 0.21	3.83 ± 0.07	3.63 ± 0.04	2.97 ± 0.22	0.054
	9 weeks	3 ± 0.1 ^a^	3.08 ± 0.28 ^a^	2.32 ± 0.1 ^b^	2.22 ± 0.04 ^b^	2.1 ± 0.17 ^b^	0.004
Valine	3 weeks	4.3 ± 0.1 ^a^	4.11 ± 0.06 ^ab^	4.27 ± 0.03 ^a^	4.04 ± 0.19 ^ab^	3.72 ± 0.16 ^b^	0.042
	6 weeks	3.92 ± 0.02	3.93 ± 0.02	4.59 ± 0.11	4.42 ± 0.32	3.78 ± 0.1	0.064
	9 weeks	4.79 ± 0.2 ^a^	4.32 ± 0.13 ^b^	4 ± 0.05 ^bc^	3.72 ± 0.06 ^c^	3.67 ± 0.24 ^c^	0.002
Isoleucine	3 weeks	4.15 ± 0.12	4.03 ± 0.21	4.13 ± 0.02	3.92 ± 0.19	3.57 ± 0.13	0.055
	6 weeks	3.78 ± 0.1	3.76 ± 0.08	4.45 ± 0.14	4.25 ± 0.38	3.63 ± 0.01	0.097
	9 weeks	4.64 ± 0.16 ^a^	4.35 ± 0.4 ^ab^	3.87 ± 0.09 ^bc^	3.63 ± 0.02 ^c^	3.53 ± 0.27 ^c^	0.004
Serine	3 weeks	6.07 ± 0.08	6.31 ± 0.12	6.51 ± 0.07	6.22 ± 0.36	5.78 ± 0.01	0.187
	6 weeks	5.84 ± 0	6.21 ± 0.02	7.28 ± 0.07	7.06 ± 0.41	6 ± 0.14	0.053
	9 weeks	6.42 ± 0.17 ^a^	6.18 ± 0.27 ^a^	5.87 ± 0.2 ^ab^	5.38 ± 0.14 ^b^	5.36 ± 0.14 ^b^	0.028
Threonine	3 weeks	3.65 ± 0.04 ^b^	3.59 ± 0.03 ^b^	4.33 ± 0.05 ^a^	3.91 ± 0.19 ^ab^	3.55 ± 0 ^b^	0.042
	6 weeks	3.56 ± 0.05 ^b^	3.69 ± 0.1 ^b^	4.39 ± 0.08 ^a^	4.32 ± 0.07 ^a^	3.51 ± 0.12 ^b^	0.016
	9 weeks	3.78 ± 0.15	3.48 ± 0.15	4.15 ± 0.02	3.55 ± 0.28	3.62 ± 0.13	0.216
Arginine	3 weeks	4.95 ± 0.1	5.18 ± 0.08	5.47 ± 0	5.26 ± 0.07	4.87 ± 0.14	0.074
	6 weeks	4.74 ± 0.1	5.27 ± 0.01	6.19 ± 0.07	5.86 ± 0.28	4.94 ± 0.11	0.062
	9 weeks	5.29 ± 0.11 ^a^	4.84 ± 0.04 ^ab^	4.38 ± 0.14 ^bc^	4.04 ± 0.08 ^c^	4.05 ± 0.07 ^c^	0.002
Glycine	3 weeks	2.37 ± 0.05	2.52 ± 0.35	2.48 ± 0.01	2.56 ± 0.25	2.37 ± 0.1	0.795
	6 weeks	2.33 ± 0	2.42 ± 0.13	2.85 ± 0.06	3.06 ± 0.49	2.69 ± 0.05	0.108
	9 weeks	2.44 ± 0.09	2.65 ± 0.62	2.16 ± 0.08	2.13 ± 0.04	2.04 ± 0.16	0.209
Phenylalanine	3 weeks	3.83 ± 0.1	3.95 ± 0.42	3.89 ± 0.06	3.79 ± 0.27	3.47 ± 0.19	0.449
	6 weeks	3.6 ± 0.08	3.66 ± 0.13	4.28 ± 0.13	4.32 ± 0.5	3.79 ± 0.14	0.156
	9 weeks	4.13 ± 0.13 ^ab^	4.3 ± 0.79 ^a^	3.56 ± 0 ^ac^	3.33 ± 0.08 ^bc^	3.15 ± 0.24 ^c^	0.045
Proline	3 weeks	2.94 ± 0.06	3.04 ± 0.34	3.02 ± 0.15	3.11 ± 0.18	2.91 ± 0.11	0.912
	6 weeks	2.87 ± 0.02	2.94 ± 0.16	3.47 ± 0.06	3.65 ± 0.47	3.07 ± 0.01	0.119
	9 weeks	3.05 ± 0.12 ^b^	3.16 ± 0.56 ^b^	4.41 ± 0.18 ^a^	4.42 ± 0.22 ^a^	4.8 ± 0.32 ^a^	0.001
Tyrosine	3 weeks	4.43 ± 0.16	4.55 ± 0.61	4.5 ± 0.04	4.65 ± 0.28	4.08 ± 0.09	0.463
	6 weeks	3.93 ± 0.09	4.08 ± 0.28	5.07 ± 0.18	5.25 ± 0.7	4.47 ± 0.2	0.070
	9 weeks	5.1 ± 0.26	5.11 ± 1.05	4.02 ± 0.23	4.13 ± 0.1	3.7 ± 0.41	0.061
Methionine	3 weeks	1.4 ± 0.02	1.46 ± 0.12	1.4 ± 0	1.45 ± 0.12	1.29 ± 0.06	0.269
	6 weeks	1.27 ± 0.03	1.28 ± 0.01	1.47 ± 0.07	1.53 ± 0.22	1.34 ± 0.1	0.151
	9 weeks	1.58 ± 0.07 ^ab^	1.66 ± 0.28 ^a^	1.35 ± 0.06 ^bc^	1.37 ± 0.03 ^ac^	1.24 ± 0.02 ^c^	0.044
Cysteine	3 weeks	0.67 ± 0.16 ^a^	0.25 ± 0.08 ^b^	0.22 ± 0.01 ^b^	0.25 ± 0.07 ^b^	0.22 ± 0 ^b^	0.001
	6 weeks	0.91 ± 0.27 ^a^	0.17 ± 0.02 ^b^	0.18 ± 0 ^b^	0.24 ± 0.11 ^b^	0.15 ± 0.01 ^b^	0.000
	9 weeks	0.35 ± 0.03	0.36 ± 0.17	0.26 ± 0.01	0.26 ± 0.03	0.29 ± 0.01	0.490
Others	3 weeks	34.13 ± 1.02	32.49 ± 0.94	29.5 ± 0.55	33.25 ± 2.67	38.3 ± 1.75	0.105
	6 weeks	37.95 ± 0.08 ^a^	33.63 ± 0.17 ^ac^	22.38 ± 0.78 ^c^	25.26 ± 3.83 ^bc^	36.97 ± 1.87 ^ab^	0.037
	9 weeks	28.8 ± 2.13 ^b^	32.35 ± 2.73 ^ab^	34.35 ± 1.66 ^ab^	39.25 ± 1.94 ^a^	38.38 ± 1.22 ^a^	0.035

Note: ^a–c^ Values within a row with different superscripts differ significantly at *p* < 0.05, while those with the same or no letter superscripts mean no significant difference (*p* > 0.05). Abbreviations: NC, normal temperature control group; NCX4, supplementing the daily diet with an additional 4 mg/kg CX under normal temperature; NCX6, supplementing the daily diet with an additional 6 mg/kg CX under normal temperature; NCX8, supplementing the daily diet with an additional 8 mg/kg CX under normal temperature; NCX10, supplementing the daily diet with an additional 10 mg/kg CX under normal temperature.

**Table 6:** Effects of dietary CX supplementation under normal temperature on lipid content of egg yolk: a comprehensive analysis of total cholesterol (TC), triglycerides (TG), high-density lipoprotein cholesterol (HDL-C), low-density lipoprotein cholesterol (LDL-C), and lecithin (LEC) over 3-week, 6-week, and 9-week periods. Data are presented as mean ± standard deviation. *n* = 6 replicates.

Item	Time	NC	NCX4	NCX6	NCX8	NCX10	*p*-Value
TG (mg/g)	3 weeks	33.71 ± 0.97	36.01 ± 0.83 ^AB^	35.92 ± 1.63 ^AB^	35.9 ± 0.98 ^B^	37.87 ± 2.73	0.521
	6 weeks	37.66 ± 2.89 ^a^	32.91 ± 0.79 ^Bab^	30.87 ± 1.41 ^Bb^	36.22 ± 1.29 ^Ba^	36.86 ± 0.62 ^a^	0.049
	9 weeks	41.26 ± 1.72	37.88 ± 0.67 ^A^	39.74 ± 1.20 ^A^	40.61 ± 0.68 ^A^	39.92 ± 2.73	0.651
	*p*-value	0.098	0.01	0.013	0.029	0.642	
TC (mg/g)	3 weeks	11.14 ± 0.60	10.83 ± 0.4 ^AB^	11.83 ± 0.91	13.7 ± 2.39	13.79 ± 2.18	0.533
	6 weeks	12.95 ± 0.81	11.18 ± 0.8 ^A^	10.88 ± 1.01	11.77 ± 0.56	12.01 ± 0.67	0.429
	9 weeks	10.82 ± 0.12	10.24 ± 0.34 ^B^	11.24 ± 0.86	9.94 ± 0.45	11.59 ± 0.66	0.257
	*p*-value	0.0834	0.035	0.775	0.258	0.520	
LEC (ng/g)	3 weeks	255.21 ± 14.50 ^B^	270.42 ± 25.65 ^B^	244.41 ± 45.52 ^B^	294.59 ± 41.91 ^B^	241.97 ± 18.82 ^B^	0.759
	6 weeks	349.27 ± 2.47 ^Ac^	409.07 ± 3.38 ^Ab^	415.43 ± 3.32 ^Ab^	409.25 ± 3.90 ^Ab^	441.06 ± 5.52 ^Aa^	0.000
	9 weeks	368.33 ± 3.38 ^Ac^	434.57 ± 3.11 ^Aa^	442.55 ± 3.43 ^Aa^	405.39 ± 2.23 ^Ab^	428.94 ± 5.02 ^Aa^	0.000
	*p*-value	0.000	0.001	0.004	0.026	0.000	
LDL-C (µmol/g)	3 weeks	33.32 ± 3.04	30.36 ± 2.57	35.53 ± 2.87 ^A^	33.18 ± 0.49 ^A^	32.04 ± 1.89	0.643
	6 weeks	27.6 ± 2.82	25.9 ± 2.03	25.72 ± 2.5 ^B^	27.47 ± 1.78 ^B^	28.69 ± 0.45	0.831
	9 weeks	25.86 ± 1.78	24.63 ± 0.52	26.23 ± 1.72 ^B^	22.68 ± 1.24 ^B^	27.09 ± 0.68	0.218
	*p*-value	0.187	0.165	0.048	0.004	0.063	
HDL-C (µmol/g)	3 weeks	9.62 ± 0.59 ^A^	8.5 ± 0.28 ^A^	8.97 ± 0.92 ^A^	9.45 ± 0.49 ^A^	8.43 ± 0.75	0.605
	6 weeks	6.09 ± 1.05 ^B^	5.64 ± 0.59 ^B^	4.61 ± 0.45 ^B^	6.16 ± 0.70 ^B^	6.5 ± 0.53	0.408
	9 weeks	6.85 ± 0.20 ^AB^	6.86 ± 0.17 ^AB^	7.91 ± 0.46 ^A^	7.42 ± 0.20 ^AB^	6.95 ± 0.67	0.309
	*p*-value	0.027	0.006	0.007	0.010	0.172	

Note: ^A,B^ Values within a column with different superscripts differ significantly at *p* < 0.05, while those with the same or no letter superscripts mean no significant difference (*p* > 0.05). ^a–c^ Values within a row with different superscripts differ significantly at *p* < 0.05, while those with the same or no letter superscripts mean no significant difference (*p* > 0.05). Abbreviations: NC, normal temperature control group; NCX4, supplementing the daily diet with an additional 4 mg/kg CX under normal temperature; NCX6, supplementing the daily diet with an additional 6 mg/kg CX under normal temperature; NCX8, supplementing the daily diet with an additional 8 mg/kg CX under normal temperature; NCX10, supplementing the daily diet with an additional 10 mg/kg CX under normal temperature.

**Table 7:** Effects of dietary CX supplementation under normal temperature on Calcium (Ca), Phosphorus (P), Vitamin B2 (Vb2) of egg yolk at 3, 6, and 9 weeks. Data are presented as mean ± standard deviation. *n* = 6 replicates.

Item	Time	NC	NCX4	NCX6	NCX8	NCX10	*p*-Value
Ca (g/kg)	3 weeks	0.5 ± 0.04	0.52 ± 0.02	0.54 ± 0.01	0.55 ± 0.03	0.57 ± 0.03	0.382
	6 weeks	0.43 ± 0.04	0.44 ± 0.04	0.51 ± 0.03	0.49 ± 0.03	0.49 ± 0.05	0.466
	9 weeks	0.56 ± 0.04	0.6 ± 0.07	0.57 ± 0.02	0.6 ± 0.03	0.65 ± 0.07	0.771
	*p*-value	0.117	0.125	0.305	0.086	0.175	
P (mg/kg)	3 weeks	0.98 ± 0.04	1.01 ± 0.01	1.02 ± 0.01	0.99 ± 0.04	0.98 ± 0.01 ^AB^	0.652
	6 weeks	0.96 ± 0.03	0.97 ± 0.06	1.06 ± 0.05	1 ± 0.02	0.95 ± 0.01 ^B^	0.183
	9 weeks	1 ± 0.05	1.06 ± 0.03	0.98 ± 0.04	0.98 ± 0.06	1 ± 0.01 ^A^	0.728
	*p*-value	0.796	0.360	0.455	0.945	0.020	
VB_2_ (mg/kg)	3 weeks	2.57 ± 0.08	2.83 ± 0.43	2.75 ± 0.06	2.51 ± 0.13	2.12 ± 0.15 ^B^	0.250
	6 weeks	2.46 ± 0.62	2.5 ± 0.39	2.74 ± 0.29	2.91 ± 0.45	2.99 ± 0.25 ^A^	0.462
	9 weeks	2.53 ± 0.25	1.92 ± 0.08	2 ± 0.54	2.21 ± 0.37	1.86 ± 0.3 ^B^	0.654
	*p*-value	0.979	0.237	0.307	0.414	0.037	

Note: ^A,B^ Values within a column with different superscripts differ significantly at *p* < 0.05, while those with the same or no letter superscripts mean no significant difference (*p* > 0.05). Abbreviations: NC, normal temperature control group; NCX4, supplementing the daily diet with an additional 4 mg/kg CX under normal temperature; NCX6, supplementing the daily diet with an additional 6 mg/kg CX under normal temperature; NCX8, supplementing the daily diet with an additional 8 mg/kg CX under normal temperature; NCX10, supplementing the daily diet with an additional 10 mg/kg CX under normal temperature.

**Table 8:** The impact of continuous high temperatures for 3, 6, and 9 weeks on the egg-laying performance of hens and the effect of continuous supplementation with varying concentrations of CX under high temperature on the egg-laying performance of hens were evaluated: a comprehensive analysis of egg-laying rate, average egg weight, average feed intake, yolk weight, and relative weight of egg yolk over 3-week, 6-week, and 9-week periods. Data are presented as mean ± standard deviation. *n* = 6 replicates (6 hens per replicate).

Item	Time	NC	HC	HCX4	HCX6	HCX8	HCX10	*p*-Value
Egg-laying rate (%)	3 weeks	47.61 ± 5.61 ^ab^	40.1 ± 0.45 ^b^	47.7 ± 1.96 ^Bab^	52.15 ± 1.84 ^Ba^	52.05 ± 1.12 ^a^	41.86 ± 1.50 ^b^	0.032
	6 weeks	55.63 ± 8.18 ^a^	31.48 ± 2.81 ^c^	42.89 ± 1.08 ^Bbc^	44.9 ± 1.84 ^Bab^	52.11 ± 1.18 ^a^	45.37 ± 1.85 ^ab^	0.011
	9 weeks	57.80 ± 3.81 ^a^	32.96 ± 2.55 ^c^	52.93 ± 1.43 ^Aab^	52.47 ± 2.47 ^Aab^	55.64 ± 0.66 ^a^	43.84 ± 1.43 ^b^	0.000
	*p*-value	0.506	0.068	0.014	0.034	0.087	0.343	
Average egg weight (g)	3 weeks	42.93 ± 1.22	43.21 ± 1.58	40.24 ± 0.87 ^B^	39.65 ± 0.72 ^B^	42.37 ± 1.38	40.90 ± 1.81	0.301
	6 weeks	42.66 ± 1.17	39.2 ± 1.23	39.97 ± 0.51 ^B^	41.48 ± 0.84 ^B^	41.49 ± 0.52	41.48 ± 1.17	0.166
	9 weeks	42.14 ± 1.97	41.88 ± 2.47	44.52 ± 1.78 ^A^	45.38 ± 1.30 ^A^	43.68 ± 0.64	45.24 ± 1.19	0.538
	*p*-value	0.930	0.318	0.026	0.003	0.276	0.097	
Average feed intake (g)	3 weeks	110.72 ± 18.62	84.19 ± 17.57	88.84 ± 18.84	87.91 ± 18.83	87.16 ± 18.93	82.24 ± 18.9	0.898
	6 weeks	102.9 ± 7.42	82.45 ± 4.81	84.39 ± 4.47	85.88 ± 5.53	91.29 ± 5.39	80.96 ± 4.87	0.124
	9 weeks	101.42 ± 8.61	88.26 ± 4.00	83.90 ± 4.38	81.94 ± 5.52	81.69 ± 2.09	80.58 ± 5.52	0.130
	*p*-value	0.506	0.927	0.946	0.936	0.841	0.994	
Yolk weight (g)	3 weeks	12.44 ± 0.57 ^Bb^	13.76 ± 0.6 ^ab^	14.06 ± 0.54 ^ab^	13.17 ± 0.38 ^Bab^	14.87 ± 0.39 ^Ba^	13.68 ± 0.39 ^Bab^	0.033
	6 weeks	15.94 ± 0.76 ^Aa^	13.65 ± 0.5 ^b^	14.59 ± 0.56 ^ab^	15.39 ± 0.39 ^Aa^	15.28 ± 0.38 ^ABa^	15.61 ± 0.48 ^Aa^	0.044
	9 weeks	14.82 ± 0.71 ^ABab^	13.44 ± 0.75 ^b^	14.39 ± 0.65 ^ab^	15.2 ± 0.82 ^ABab^	16.2 ± 0.18 ^Aa^	15.21 ± 0.34 ^Aab^	0.048
	*p*-value	0.008	0.935	0.810	0.026	0.036	0.010	
Relative weight of egg yolk (%)	3 weeks	29.10 ± 1.65 ^Bb^	31.88 ± 1.11 ^ab^	34.94 ± 1.12 ^ABa^	33.19 ± 0.47 ^Bab^	35.27 ± 1.42 ^a^	33.83 ± 2.06 ^ab^	0.041
	6 weeks	37.49 ± 1.95 ^A^	34.86 ± 1.00	36.47 ± 1.08 ^A^	37.18 ± 1.16 ^A^	36.81 ± 0.48	37.65 ± 0.81	0.586
	9 weeks	35.26 ± 1.26 ^Aa^	32.15 ± 0.53 ^b^	32.3 ± 0.43 ^Bb^	33.42 ± 1.22 ^Bab^	37.11 ± 0.3 ^a^	33.72 ± 0.99 ^ab^	0.002
	*p*-value	0.007	0.069	0.018	0.017	0.313	0.121	

Note: ^A,B^ Values within a column with different superscripts differ significantly at *p* < 0.05, while those with the same or no letter superscripts mean no significant difference (*p* > 0.05). ^a–c^ Values within a row with different superscripts differ significantly at *p* < 0.05, while those with the same or no letter superscripts mean no significant difference (*p* > 0.05). Abbreviations: NC, normal temperature control group; HC, high-temperature control group; HCX4, supplementing the daily diet with an additional 4 mg/kg CX under high temperature; HCX6, supplementing the daily diet with an additional 6 mg/kg CX under high temperature; HCX8, supplementing the daily diet with an additional 8 mg/kg CX under high temperature; HCX10, supplementing the daily diet with an additional 10 mg/kg CX under high temperature.

**Table 9:** The impact of continuous high temperatures for 3, 6, and 9 weeks on total amino acid content of egg yolks and the effect of continuous supplementation with varying concentrations of CX for 3, 6, and 9 weeks under high temperature on total amino acid content of egg yolks were evaluated. Data are presented as mean ± standard deviation. *n* = 6 replicates.

Total Amino Acid Content (μmol/g)	3 Weeks	6 Weeks	9 Weeks	*p*-Value
NC	123.99 ± 1.13 ^ABa^	137.24 ± 4.42 ^Aa^	110.75 ± 5.66 ^B^	0.012
HC	114.65 ± 2.40 ^b^	117.54 ± 4.30 ^b^	111.75 ± 9.10	0.798
HCX4	111.88 ± 1.34 ^b^	112.11 ± 2.36 ^b^	111.65 ± 2.50	0.988
HCX6	114.8 ± 3.98 ^b^	113.14 ± 5.63 ^b^	116.46 ± 2.38	0.86
HCX8	112.27 ± 3.08 ^b^	107.65 ± 1.27 ^b^	116.88 ± 5.38	0.279
HCX10	116.58 ± 3.33 ^ab^	115 ± 1.09 ^b^	118.16 ± 6.80	0.882
*p*-value	0.042	0.001	0.897	

Note: ^A,B^ Values within a column with different superscripts differ significantly at *p* < 0.05, while those with the same or no letter superscripts mean no significant difference (*p* > 0.05). ^a,b^ Values within a row with different superscripts differ significantly at *p* < 0.05, while those with the same or no letter superscripts mean no significant difference (*p* > 0.05). Abbreviations: NC, normal temperature control group; HC, high-temperature control group; HCX4, supplementing the daily diet with an additional 4 mg/kg CX under high temperature; HCX6, supplementing the daily diet with an additional 6 mg/kg CX under high temperature; HCX8, supplementing the daily diet with an additional 8 mg/kg CX under high temperature; HCX10, supplementing the daily diet with an additional 10 mg/kg CX under high temperature.

**Table 10:** The impact of continuous high temperatures for 3, 6, and 9 weeks on percentages of 17 individual amino acids of egg yolks and the effect of continuous supplementation for 3, 6, and 9 weeks with varying concentrations of CX under high temperature on percentages of 17 individual amino acids of egg yolks were evaluated. Data are presented as mean ± standard deviation. *n* = 6 replicates.

Proportion (%)	Time	HC	HCX4	HCX6	HCX8	HCX10	*p*-Value
Leucine	3 weeks	8.24 ± 0.03	7.82 ± 0.13	7.85 ± 0.18	7.82 ± 0.2	7.48 ± 0.34	0.376
	6 weeks	8.64 ± 0.18	8.29 ± 0.26	8.42 ± 0.25	8.52 ± 0.25	7.67 ± 0.03	0.245
	9 weeks	7.95 ± 0.17	7.35 ± 0.02	7.32 ± 0.12	7.16 ± 0.13	7.31 ± 0.69	0.442
Asparagine	3 weeks	5.02 ± 0.26	4.9 ± 0.05	5.03 ± 0.23	4.89 ± 0.04	4.32 ± 0.15	0.272
	6 weeks	4.25 ± 0.18	3.96 ± 0.35	4.41 ± 0.56	4.31 ± 0.76	4.03 ± 0.19	0.518
	9 weeks	5.86 ± 0.3	5.88 ± 0.27	5.65 ± 0.13	5.46 ± 0.62	4.64 ± 0.5	0.383
Histidine	3 weeks	1.6 ± 0.04	1.47 ± 0.02	1.48 ± 0.04	1.45 ± 0.04	1.42 ± 0.07	0.250
	6 weeks	1.88 ± 0.03	1.76 ± 0.05	1.8 ± 0.07	1.76 ± 0.06	1.62 ± 0.01	0.462
	9 weeks	1.34 ± 0.08	1.19 ± 0	1.17 ± 0.02	1.15 ± 0.02	1.23 ± 0.12	0.089
Lysine	3 weeks	6.87 ± 0.04	6.04 ± 0.22	6.41 ± 0.27	6.02 ± 0	5.49 ± 0.11	0.093
	6 weeks	6.91 ± 0.24	6.01 ± 0.5	6.89 ± 0.49	6.5 ± 0.41	6.05 ± 0.18	0.374
	9 weeks	6.9 ± 0.12 ^a^	6.08 ± 0.07 ^ab^	5.97 ± 0.06 ^ab^	5.58 ± 0.34 ^ab^	4.95 ± 0.34 ^b^	0.015
Glutamic acid	3 weeks	4.95 ± 0.21	5.17 ± 0.25	5.3 ± 0.41	4.82 ± 0.01	4.17 ± 0.12	0.096
	6 weeks	5.12 ± 0.33	5.06 ± 0.5	5.77 ± 0.8	5.5 ± 0.62	5.04 ± 0.49	0.459
	9 weeks	4.81 ± 0.12 ^ab^	5.32 ± 0 ^a^	4.87 ± 0.02 ^ab^	4.22 ± 0.49 ^bc^	3.34 ± 0.15 ^c^	0.011
Alanine	3 weeks	3.4 ± 0.03	2.9 ± 0.05	3.02 ± 0.22	2.88 ± 0.12	2.92 ± 0.1	0.085
	6 weeks	3.71 ± 0.05	3.42 ± 0.04	3.65 ± 0.37	3.38 ± 0.22	3.09 ± 0.22	0.532
	9 weeks	3.08 ± 0.04 ^a^	2.38 ± 0.06 ^b^	2.42 ± 0.09 ^b^	2.41 ± 0.05 ^b^	2.77 ± 0.39 ^ab^	0.019
Valine	3 weeks	4.59 ± 0.02 ^a^	4.14 ± 0.04 ^bc^	3.91 ± 0.14 ^bd^	3.81 ± 0.04 ^cd^	3.57 ± 0.13 ^d^	0.001
	6 weeks	4.6 ± 0.09 ^a^	4.06 ± 0.09 ^ab^	3.9 ± 0.18 ^b^	3.91 ± 0.05 ^b^	3.45 ± 0.03 ^b^	0.040
	9 weeks	4.65 ± 0.11 ^ab^	4.22 ± 0.01 ^ac^	3.92 ± 0.09 ^bc^	3.73 ± 0.11 ^c^	3.7 ± 0.3 ^c^	0.019
Isoleucine	3 weeks	4.36 ± 0 ^a^	3.97 ± 0.08 ^ac^	3.87 ± 0.08 ^bc^	3.87 ± 0.08 ^bc^	3.71 ± 0.2 ^c^	0.031
	6 weeks	4.3 ± 0.08	3.9 ± 0.18	3.87 ± 0.06	3.96 ± 0.1	3.49 ± 0.07	0.386
	9 weeks	4.51 ± 0.07	4.03 ± 0.02	3.88 ± 0.09	3.78 ± 0.06	3.94 ± 0.37	0.061
Serine	3 weeks	6.76 ± 0.05	6.35 ± 0.04	6.37 ± 0.12	6.17 ± 0.24	5.79 ± 0.27	0.078
	6 weeks	7.14 ± 0.15	6.7 ± 0.05	7 ± 0.2	6.83 ± 0.18	6.21 ± 0.02	0.198
	9 weeks	6.46 ± 0.19	6.01 ± 0.02	5.77 ± 0.04	5.56 ± 0.26	5.4 ± 0.51	0.159
Threonine	3 weeks	3.93 ± 0.06	3.94 ± 0.06	3.97 ± 0.14	3.8 ± 0.07	3.41 ± 0.13	0.143
	6 weeks	4.29 ± 0.12	3.91 ± 0.03	4.11 ± 0.26	3.95 ± 0.21	3.52 ± 0.13	0.146
	9 weeks	3.61 ± 0.2	3.99 ± 0.15	3.85 ± 0.01	3.68 ± 0.31	3.31 ± 0.36	0.600
Arginine	3 weeks	5.61 ± 0.02 ^a^	4.88 ± 0.05 ^b^	5 ± 0.17 ^b^	4.76 ± 0.17 ^b^	4.4 ± 0.24 ^b^	0.023
	6 weeks	5.92 ± 0.15	5.5 ± 0.08	5.88 ± 0.33	5.65 ± 0.01	5.05 ± 0.03	0.213
	9 weeks	5.36 ± 0.15 ^a^	4.26 ± 0.02 ^b^	4.17 ± 0.03 ^b^	3.94 ± 0.26 ^b^	3.77 ± 0.43 ^b^	0.004
Glycine	3 weeks	2.61 ± 0.03	2.75 ± 0.13	2.6 ± 0.04	2.67 ± 0.13	2.63 ± 0.17	0.257
	6 weeks	2.89 ± 0.03	3.14 ± 0.3	2.93 ± 0.02	3.04 ± 0.3	2.72 ± 0.01	0.272
	9 weeks	2.38 ± 0.05	2.35 ± 0.04	2.28 ± 0.07	2.33 ± 0.03	2.54 ± 0.32	0.728
Phenylalanine	3 weeks	4.06 ± 0.03	3.98 ± 0.2	3.81 ± 0.05	3.92 ± 0.15	3.76 ± 0.22	0.643
	6 weeks	4.26 ± 0.07	4.24 ± 0.41	4.03 ± 0.01	4.21 ± 0.3	3.69 ± 0.03	0.360
	9 weeks	3.92 ± 0.03	3.72 ± 0.02	3.61 ± 0.12	3.63 ± 0.01	3.84 ± 0.41	0.348
Proline	3 weeks	3.19 ± 0.04 ^c^	4.36 ± 0.25 ^a^	4.32 ± 0.03 ^ab^	4.21 ± 0.4 ^ab^	3.61 ± 0.06 ^bc^	0.002
	6 weeks	3.49 ± 0.04	3.57 ± 0.24	3.41 ± 0.13	3.29 ± 0.37	2.81 ± 0.05	0.350
	9 weeks	2.94 ± 0.07 ^b^	5.14 ± 0.25 ^a^	5.21 ± 0.12 ^a^	5.07 ± 0.46 ^a^	4.4 ± 0.25 ^a^	0.000
Tyrosine	3 weeks	4.88 ± 0.02	4.47 ± 0.16	4.45 ± 0.01	4.71 ± 0.12	4.8 ± 0.5	0.378
	6 weeks	5.11 ± 0.08	4.88 ± 0.34	4.78 ± 0.09	4.93 ± 0.36	4.4 ± 0.4	0.259
	9 weeks	4.74 ± 0.08 ^ab^	4.06 ± 0.02 ^b^	4.14 ± 0.06 ^b^	4.5 ± 0.1 ^ab^	5.21 ± 0.64 ^a^	0.050
Methionine	3 weeks	1.49 ± 0.05	1.44 ± 0.04	1.45 ± 0.02	1.47 ± 0.11	1.54 ± 0.14	0.642
	6 weeks	1.5 ± 0.04	1.5 ± 0.11	1.48 ± 0.02	1.48 ± 0.23	1.4 ± 0.02	0.663
	9 weeks	1.52 ± 0.11	1.38 ± 0.04	1.42 ± 0.06	1.45 ± 0	1.67 ± 0.28	0.369
Cysteine	3 weeks	0.36 ± 0.06 ^b^	0.3 ± 0.08 ^b^	0.26 ± 0.01 ^b^	0.25 ± 0 ^b^	0.27 ± 0.01 ^b^	0.001
	6 weeks	0.44 ± 0.12 ^b^	0.28 ± 0.14 ^b^	0.21 ± 0.01 ^b^	0.2 ± 0.02 ^b^	0.2 ± 0.03 ^b^	0.000
	9 weeks	0.28 ± 0	0.33 ± 0.01	0.31 ± 0	0.29 ± 0.01	0.33 ± 0.06	0.214
Others	3 weeks	28.07 ± 0.17	31.1 ± 0.71	30.89 ± 2.13	32.43 ± 1.59	36.64 ± 2.71	0.234
	6 weeks	25.55 ± 1.74	29.82 ± 0.84	27.48 ± 3.6	28.5 ± 0.12	35.46 ± 0.86	0.265
	9 weeks	29.69 ± 0.93	32.33 ± 0.57	34 ± 0.66	35.99 ± 2.93	37.55 ± 6.13	0.472

Note: ^a–d^ Values within a row with different superscripts differ significantly at *p* < 0.05, while those with the same or no letter superscripts mean no significant difference (*p* > 0.05). Abbreviations: NC, normal temperature control group; HC, high-temperature control group; HCX4, supplementing the daily diet with an additional 4 mg/kg CX under high temperature; HCX6, supplementing the daily diet with an additional 6 mg/kg CX under high temperature; HCX8, supplementing the daily diet with an additional 8 mg/kg CX under high temperature; HCX10, supplementing the daily diet with an additional 10 mg/kg CX under high temperature.

**Table 11:** The impact of high temperatures on lipid content of egg yolk and the effects of dietary CX supplementation under high temperature on lipid content of egg yolk: a comprehensive analysis of total cholesterol (TC), triglycerides (TG), high-density lipoprotein cholesterol (HDL-C), low-density lipoprotein cholesterol (LDL-C), and lecithin (LEC) over 3-week, 6-week, and 9-week periods. Data are presented as mean ± standard deviation. *n* = 6 replicates.

Item	Time	NC	HC	HCX4	HCX6	HCX8	HCX10	*p*-Value
TG (mg/g)	3 weeks	33.71 ± 0.97 ^b^	37.24 ± 1.34 ^Bab^	36.95 ± 1.09 ^Aab^	38.36 ± 0.24 ^a^	40.08 ± 1.73 ^a^	40.91 ± 1.92 ^a^	0.029
	6 weeks	37.66 ± 2.89	32.77 ± 1.23 ^C^	33.00 ± 0.29 ^B^	36.04 ± 0.76	36.86 ± 1.52	36.98 ± 1.11	0.519
	9 weeks	41.26 ± 1.72	40.62 ± 1.80 ^A^	39.07 ± 0.03 ^A^	38.33 ± 1.66	41.32 ± 0.06	42.30 ± 2.51	0.518
	*p*-value	0.098	0.026	0.002	0.283	0.125	0.211	
TC (mg/g)	3 weeks	11.14 ± 0.60 ^c^	12.07 ± 0.49 ^bc^	14.18 ± 0.44 ^ab^	12.16 ± 0.69 ^bc^	14.84 ± 0.76 ^a^	13.43 ± 0.18 ^ac^	0.004
	6 weeks	12.95 ± 0.81	12.39 ± 0.30	14.39 ± 1.34	13.84 ± 1.73	15.21 ± 1.53	14.09 ± 0.92	0.110
	9 weeks	10.82 ± 0.12 ^b^	12.39 ± 0.30 ^a^	11.63 ± 0.32 ^ab^	12.51 ± 0.86 ^a^	11.71 ± 0.70 ^ab^	12.76 ± 0.11 ^a^	0.045
	*p*-value	0.8	0.796	0.104	0.597	0.11	0.298	
LEC (ng/g)	3 weeks	255.21 ± 14.50 ^B^	226.14 ± 17.91 ^B^	285.92 ± 43.39 ^B^	264.98 ± 34.02 ^B^	274.87 ± 38.35 ^B^	270.61 ± 47.30 ^B^	0.870
	6 weeks	349.27 ± 2.47 ^Ac^	340.55 ± 3.07 ^Ab^	407.93 ± 1.81 ^Aa^	428.56 ± 3.42 ^Aa^	422.1 ± 5.41 ^Aa^	427.76 ± 7.80 ^Aa^	0.000
	9 weeks	368.33 ± 3.38 ^Ab^	333.34 ± 6.59 ^Ac^	422.34 ± 2.14 ^Aa^	409.42 ± 5.06 ^Aa^	412.82 ± 3.06 ^Aa^	410.12 ± 5.25 ^Aa^	0.000
	*p*-value	0.000	0.001	0.016	0.002	0.006	0.014	
LDL-C (µmol/g)	3 weeks	33.32 ± 3.04	34.45 ± 2.17	30.81 ± 0.96	32.45 ± 1.16 ^A^	33.39 ± 1.42	32.07 ± 1.31	0.787
	6 weeks	27.6 ± 2.82	26.26 ± 3.85	26.08 ± 3.16	28.55 ± 1.16 ^AB^	29.44 ± 2.6	31.38 ± 2.36	0.753
	9 weeks	25.86 ± 1.78 ^b^	29.53 ± 0.8 ^a^	29.64 ± 0.47 ^a^	27.44 ± 0.79 ^Bab^	25.35 ± 0.81 ^b^	28.52 ± 0.7 ^ab^	0.036
	*p*-value	0.187	0.160	0.272	0.034	0.050	0.325	
HDL-C (µmol/g)	3 weeks	9.62 ± 0.59 ^A^	9.92 ± 0.49 ^A^	9.79 ± 0.52 ^A^	10.28 ± 0.34 ^A^	10.04 ± 0.16 ^A^	10.04 ± 0.16 ^A^	0.901
	6 weeks	6.09 ± 1.05 ^Bb^	6.43 ± 1.03 ^Bb^	6.9 ± 0.65 ^Bab^	7.22 ± 0.43 ^Bab^	8.32 ± 0.78 ^ABab^	9.12 ± 0.54 ^ABa^	0.042
	9 weeks	6.85 ± 0.20 ^Bb^	6.81 ± 0.41 ^Bb^	6.99 ± 0.12 ^Bb^	7.37 ± 0.21 ^Bb^	7.54 ± 0.17 ^Bab^	8.57 ± 0.05 ^Ba^	0.001
	*p*-value	0.027	0.023	0.009	0.001	0.023	0.048	

Note: ^A–C^ Values within a column with different superscripts differ significantly at *p* < 0.05, while those with the same or no letter superscripts mean no significant difference (*p* > 0.05). ^a–c^ Values within a row with different superscripts differ significantly at *p* < 0.05, while those with the same or no letter superscripts mean no significant difference (*p* > 0.05). Abbreviations: NC, normal temperature control group; HC, high-temperature control group; HCX4, supplementing the daily diet with an additional 4 mg/kg CX under high temperature; HCX6, supplementing the daily diet with an additional 6 mg/kg CX under high temperature; HCX8, supplementing the daily diet with an additional 8 mg/kg CX under high temperature; HCX10, supplementing the daily diet with an additional 10 mg/kg CX under high temperature.

**Table 12:** The impact of high temperatures on lipid content of egg yolk and the effects of dietary CX supplementation under high temperature on lipid content of egg yolk: a comprehensive analysis of Calcium (Ca), Phosphorus (P), Vitamin B_2_ (Vb_2_) over 3-week, 6-week, and 9-week periods. Data are presented as mean ± standard deviation. *n* = 6 replicates.

Item	Time	NC	HC	HCX4	HCX6	HCX8	HCX10	*p*-Value
Ca (g/kg)	3 weeks	0.5 ± 0.04 ^c^	0.53 ± 0.00 ^c^	0.52 ± 0.00 ^Bc^	0.6 ± 0.03 ^a^	0.57 ± 0.02 ^ab^	0.61 ± 0.01 ^Ba^	0.015
	6 weeks	0.43 ± 0.04 ^b^	0.53 ± 0.02 ^a^	0.48 ± 0.01 ^Cab^	0.54 ± 0.02 ^a^	0.54 ± 0 ^a^	0.54 ± 0.03 ^Ca^	0.016
	9 weeks	0.56 ± 0.04 ^c^	0.53 ± 0.01 ^c^	0.57 ± 0.01 ^Abc^	0.67 ± 0.06 ^ab^	0.61 ± 0.03 ^ac^	0.69 ± 0.01 ^Aa^	0.026
	*p*-value	0.117	0.991	0.000	0.153	0.150	0.005	
P (mg/kg)	3 weeks	0.98 ± 0.04	1.01 ± 0.02	0.98 ± 0.02	0.99 ± 0.02	1.01 ± 0.02	0.99 ± 0.02	0.792
	6 weeks	0.96 ± 0.03	1.05 ± 0.03	1.01 ± 0.03	1 ± 0.04	0.98 ± 0.04	0.97 ± 0.04	0.622
	9 weeks	1.00 ± 0.05	0.98 ± 0.00	0.94 ± 0.00	0.98 ± 0.01	1.04 ± 0.01	1.00 ± 0.02	0.132
	*p*-value	0.797	0.129	0.100	0.895	0.370	0.764	
VB_2_ (mg/kg)	3 weeks	2.57 ± 0.08 ^a^	1.86 ± 0.1 ^Abc^	2.24 ± 0.11 ^ac^	1.78 ± 0.09 ^c^	2.34 ± 0.21 ^ab^	2.3 ± 0.26 ^ab^	0.030
	6 weeks	2.46 ± 0.62 ^ab^	1.3 ± 0.16 ^Bb^	1.64 ± 0.39 ^ab^	2.97 ± 0.57 ^a^	2.81 ± 0.41 ^a^	2.51 ± 0.37 ^a^	0.016
	9 weeks	2.53 ± 0.25 ^ab^	1.08 ± 0.06 ^Bb^	1.45 ± 0.30 ^b^	3.64 ± 0.66 ^a^	2.18 ± 0.27 ^ab^	3.11 ± 0.82 ^a^	0.018
	*p*-value	0.979	0.006	0.213	0.099	0.380	0.576	

Note: ^A–C^ Values within a column with different superscripts differ significantly at *p* < 0.05, while those with the same or no letter superscripts mean no significant difference (*p* > 0.05). ^a–c^ Values within a row with different superscripts differ significantly at *p* < 0.05, while those with the same or no letter superscripts mean no significant difference (*p* > 0.05). Abbreviations: NC, normal temperature control group; HC, high-temperature control group; HCX4, supplementing the daily diet with an additional 4 mg/kg CX under high temperature; HCX6, supplementing the daily diet with an additional 6 mg/kg CX under high temperature; HCX8, supplementing the daily diet with an additional 8 mg/kg CX under high temperature; HCX10, supplementing the daily diet with an additional 10 mg/kg CX under high temperature.

## Data Availability

The original contributions presented in the study are included in the article/Supplementary Materials. Further inquiries can be directed to the corresponding author.

## Supplementary Materials

The following supporting information can be downloaded at: https://www.mdpi.com/article/10.3390/foods14060950/s1, Figure S1. Environmental data in the process of the experiment. (A) Daily temperature records during the experiment period; (B) Daily relative humidity records throughout the experimentation period; Table S1. The statistics of mortality rate during the experimentation period.
