# Supplementary material for: Effect of Canthaxanthin on Egg Yolk Quality of Huaixiang Laying Hens at Normal and High Temperature
Source: Foods. 2025 Mar 11;14(6):950. doi: 10.3390/foods14060950 (PMC11941421; doi:10.3390/foods14060950)
Supplement: Supplementary file 1 [file foods-14-00950-s001.zip › Supplementary Material - figure.pdf]

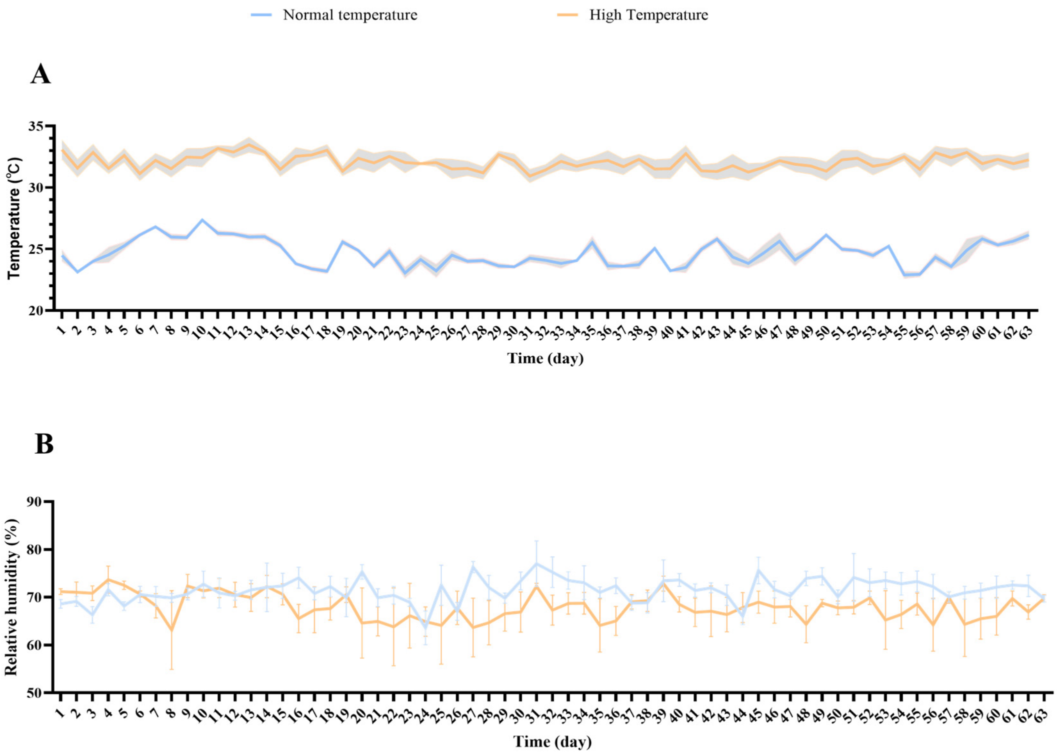

Figure S1. Environmental data in the process of the experiment. (**A**)Daily temperature records during the experiment period;(**B**)Daily relative humidity records throughout the experimentation period
