# Supplementary material for: Effect of Canthaxanthin on Egg Yolk Quality of Huaixiang Laying Hens at Normal and High Temperature
Source: Foods. 2025 Mar 11;14(6):950. doi: 10.3390/foods14060950 (PMC11941421; doi:10.3390/foods14060950)
Supplement: Supplementary file 1 [file foods-14-00950-s001.zip › Supplementary Materials - Tables.pdf]

Table S1. The statistics of mortality rate during the experimentation period.

Item	Time	NC	NT1	NT2	NT3	NT4	HC	HT1	HT2	HT3	HT4
Mortality rate (%)	0-3 Weeks	0	0	0	0	0	8.33	2.78	0	0	2.78
	3-6 Weeks	0	0	3.33	0	3.33	3.7	3.45	0	0	0
	6-9 Weeks	0	0	0	0	4.35	5.00	4.55	4.35	0	0
